# Development and comparative evaluation of machine learning algorithms and Cox regression for predicting fasting plasma glucose-defined incident prediabetes: a longitudinal cohort study

**DOI:** 10.3389/fendo.2026.1862258

**Published:** 2026-09-24

**Authors:** Su Hu, Changshun Yan, Xinyuan Tian, Menghan Xu, Bo Cheng, Tong Xu, Guiqiu Cao

**Affiliations:** 1 Department of Cardiology, the Fifth Affiliated Hospital of Xinjiang Medical University, Urumqi, China; 2 Department of Toxicology and Cancer Biology, University of Kentucky, Lexington, KY, United States

**Keywords:** diabetes mellitus, machine learning, online risk calculator, prediabetes, SHAPley additive explanations

## Abstract

**Objective:**

To develop, compare, and externally evaluate machine learning (ML) and Cox regression models for predicting fasting plasma glucose (FPG)-defined incident prediabetes.

**Methods:**

We performed a secondary analysis of a publicly available Chinese health-examination cohort and conducted an external comparative evaluation in a separate hospital-based cohort. The development cohort of adults with normal baseline fasting glucose was divided into training and internal validation sets. Candidate predictors were selected using least absolute shrinkage and selection operator (LASSO) regression. Six ML models and an initial proportional-hazards Cox model were compared using discrimination and threshold-based metrics. After identifying FPG nonproportionality, four Cox specifications were compared. Model selection considered fit, performance in the internal validation set and external cohort, complexity, and parsimony. The final model was evaluated at the primary 3-, 4-, and 5-year horizons using time-dependent area under the receiver operating characteristic curve (AUC), Brier scores, calibration, and decision curve analysis. SHapley Additive exPlanations (SHAP), sensitivity analyses, and an online calculator supported interpretation and implementation.

**Results:**

Among 10, 592 participants, 1, 287 (12.2%) developed FPG-defined incident prediabetes over a mean follow-up of 3.05 years. LASSO retained 11 of 17 predictors. Random forest showed the highest apparent training performance, but its performance declined in the internal validation set and external cohort, whereas the initial Cox model demonstrated more consistent discrimination. FPG violated the proportional-hazards assumption. The extended Cox model incorporating FPG × log(t/3) (M2) had the lowest Akaike information criterion, accommodated the time-varying FPG effect, and performed comparably to more complex alternatives; it was therefore selected. M2 retained age, body mass index, diastolic blood pressure, FPG, and family history of diabetes. Its 3- and 4-year AUCs were 0.800 and 0.796 in training, 0.778 and 0.755 in internal validation, and 0.750 and 0.764 in the external cohort. Five-year performance was exploratory and less stable externally. SHAP identified FPG as the dominant contributor, and sensitivity analyses generally supported robustness.

**Conclusion:**

The final extended Cox model (M2) and online risk calculator may provide a practical framework for interpretable, individualized risk assessment of FPG-defined incident prediabetes, supporting early risk stratification and preventive risk-factor management using routinely available clinical indicators.

## Introduction

According to the American Diabetes Association (ADA), prediabetes has two subtypes: impaired fasting glucose, with fasting plasma glucose (FPG) between 5.6-6.9 mmol/L, and impaired glucose tolerance (IGT), with 2-hour post-75g glucose load levels of 7.8-11.1 mmol/L (1, 2). The global prevalence of prediabetes is rising. In 2017, about 374 million adults had prediabetes, and this number is projected to increase to 548 million by 2045 (3). Globally, 5-10% of prediabetes patients develop diabetes annually. Prediabetes is closely linked to increased risks of nephropathy, chronic kidney disease, small-fiber neuropathy, and diabetic retinopathy (4). Prediabetes also significantly increases the risk of cardiovascular disease and all-cause mortality. The average life expectancy of people with prediabetes is 0.7 years shorter than those with normoglycemia (5). The progression from normal glucose tolerance to prediabetes is associated with various factors, including geographical and environmental factors, unhealthy lifestyles, and biological factors such as obesity, hypertension, and dyslipidemia (2, 6). Early identification, intervention in unhealthy lifestyles, and control of risk factors can effectively prevent the progression of prediabetes to diabetes (2). Thus, there is an urgent need to develop clinical tools to identify high-risk prediabetes individuals in the normal glucose population. Identifying risk factors helps create personalized intervention strategies and prevents excessive intervention in low-risk groups.

With the advancement of science and technology, artificial intelligence (AI) has played a significant role in various fields (7), especially in medical diagnosis, where it offers new insights and approaches. Machine learning (ML), a branch of AI, extracts potential and meaningful rules from large datasets using various algorithms. It has been widely applied in predictive and diagnostic models for cardiovascular diseases, cancers, and endocrine metabolic disorders (8, 9). Xue et al. (10) applied decision tree, random forest (RF), adaptive boosting (AdaBoost), and eXtreme Gradient Boosting (XGBoost) classifiers to identify potential type 2 diabetes mellitus (T2DM) in a large-scale Chinese population, and XGBoost achieved the best predictive performance, with an area under the curve (AUC) of 0.968. New intelligent diagnostic algorithms are emerging in large numbers, providing strong support for the early detection, precise diagnosis of clinical diseases, and the development of personalized treatment plans. However, ML models have certain limitations, such as their high dependence on data quality, insufficient model interpretability, and complex application in clinical practice (11). In this context, the traditional Cox regression model remains a common method for predicting disease prognosis in medical research due to its advantages in handling survival data and its clear interpretation of individual variable impacts (11, 12). Thus, constructing a simple and clinical prediabetes predictive model via ML or traditional survival analysis is of great significance.

Prediction models for FPG-defined incident prediabetes among adults with normal fasting glucose remain limited, particularly in large Chinese populations with external comparative evaluation in a separate clinical cohort. We therefore conducted a secondary analysis of a publicly available large-scale cohort study of Chinese adults and used a separate hospital-based cohort from the Fifth Affiliated Hospital of Xinjiang Medical University for external comparative evaluation (13). Using ML algorithms and Cox regression, we aimed to develop and compare prediction models for FPG-defined incident prediabetes among Chinese adults with normal fasting glucose. By comparing predictive performance across models and evaluation cohorts, while considering interpretability, parsimony, and clinical simplicity, we sought to identify a final model for individualized 3- and 4-year risk estimation of recorded FPG-defined incident prediabetes, with 5-year prediction considered exploratory, and to provide an interpretable basis for future risk-stratification and prevention studies.

## Materials and methods

### Definitions

The primary outcome was FPG-defined incident prediabetes. Among participants with normal fasting glucose at baseline, incident prediabetes was defined as a recorded follow-up FPG level of 5.6 to <7.0 mmol/L, according to the ADA criterion for impaired fasting glucose (1, 2). Follow-up time was calculated from baseline to the recorded follow-up examination at which FPG-defined incident prediabetes was identified or to the last available follow-up visit for participants who remained event-free.

### Development cohort and participant selection

The development cohort was based on a publicly available Dryad dataset shared by Chen et al. in BMJ Open in the article entitled “Association of body mass index and age with incident diabetes in Chinese adults: a population-based cohort study” (13). The original cohort was established by Rich Healthcare Group in China and included adults aged ≥20 years who underwent health examinations at least twice between 2010 and 2016 across 32 centers in 11 Chinese cities, including Beijing, Shanghai, Guangzhou, Shenzhen, Nanjing, Wuhan, Chengdu, Suzhou, Changzhou, Hefei, and Nantong. At each visit, demographic characteristics, lifestyle factors, medical history, and family history of chronic diseases were collected using standardized questionnaires. Trained staff measured height, weight, and blood pressure, and body mass index (BMI) was calculated as weight in kilograms divided by height in meters squared. Fasting venous blood samples were collected after at least 10 hours of fasting, and biochemical measurements, including FPG and lipid profiles, were performed using standardized laboratory methods.

In the original study, 685, 277 participants were initially eligible. Participants were excluded if they had missing height or weight measurements (n=103, 946), missing sex information (n=1), extreme BMI values (<15 or >55 kg/m²; n=152), missing FPG data (n=31, 370), a visit interval of less than 2 years (n=324, 233), diabetes at baseline defined by self-reported diabetes or FPG ≥7.0 mmol/L (n=7, 112), or undefined diabetes status during follow-up (n=6, 630). After these exclusions, 211, 833 participants without diabetes at baseline remained in the original analytic cohort. For the present analysis, we further restricted the cohort to participants with normal fasting glucose at baseline to evaluate FPG-defined incident prediabetes. To ensure complete-case analysis and harmonized predictor availability, participants were excluded if they had missing systolic blood pressure (SBP) or diastolic blood pressure (DBP) measurements (n=24), missing cholesterol or triglyceride data (n=4, 887), missing high-density lipoprotein (HDL) or low-density lipoprotein (LDL) data (n=93, 421), missing alanine aminotransferase (ALT) or aspartate aminotransferase (AST) data (n=29, 869), missing blood urea nitrogen (BUN) or creatinine data (n=21, 551), missing smoking or drinking history (n=28, 313), or baseline FPG ≥5.6 mmol/L or final follow-up FPG in the diabetic range (≥7.0 mmol/L) (n=23, 176). Ultimately, 10, 592 participants were included in the development cohort for secondary analysis.

### External comparative evaluation cohort

A separate hospital-based cohort used for external comparative evaluation was derived from the hospital information system (HIS) of the Fifth Affiliated Hospital of Xinjiang Medical University and included hospital-based patients with baseline records between 2016 and 2021 and follow-up data available through December 2025. To enhance comparability with the development cohort, eligibility criteria were harmonized with those of the original cohort as closely as possible. Eligible participants were aged ≥20 years, had no diabetes at baseline, had an initial FPG level <5.6 mmol/L, had at least one follow-up FPG measurement, and had a follow-up interval of more than 2 years. Participants were required to have complete demographic, clinical, and laboratory data at baseline and follow-up. We excluded participants with previously diagnosed diabetes, use of glucose-lowering medications, missing key variables, extreme BMI values (<15 or >55 kg/m²), unclear follow-up glycemic status, or clinically implausible laboratory values. To minimize the influence of acute illness or stress-related hyperglycemia, patients from emergency departments, intensive care units, perioperative settings, acute infection episodes, periods of glucocorticoid therapy, or with severe wasting disease or active malignancy were excluded when identifiable from hospital records. Ultimately, 671 patients were included. Baseline demographic, lifestyle, anthropometric, blood pressure, and laboratory data were extracted from standardized hospital records. Biochemical measurements were performed using peripheral venous blood samples collected after at least 10 hours of fasting and analyzed according to routine hospital laboratory protocols.

### Ethical approval

The development cohort was derived from a previously published retrospective cohort study using a publicly available, de-identified dataset from Dryad. Ethical approval and informed consent procedures for the original cohort were reported in the original publication (13). The use of clinical data from the external cohort was approved by the Ethics Committee of the Fifth Affiliated Hospital of Xinjiang Medical University (approval No. XYDWFYLSk-2026-33). The requirement for informed consent for the external cohort was waived because of the retrospective design and the use of de-identified clinical data.

### Machine learning algorithms

Six ML algorithms were developed and compared: RF, gradient boosting decision tree (GBDT), XGBoost, naive Bayes (NB), AdaBoost, and support vector machine (SVM). Model performance was evaluated within a time-to-event framework using time-dependent discrimination metrics to account for right-censored follow-up data (14). Briefly, RF and GBDT are tree-based ensemble methods that capture nonlinear associations and variable interactions, XGBoost and AdaBoost are boosting algorithms that iteratively improve predictive performance by focusing on poorly predicted observations, NB estimates outcome probabilities based on Bayes’ theorem under a conditional independence assumption, and SVM identifies an optimal separating hyperplane after mapping predictors into a higher-dimensional feature space (14–19). The modeling framework, preprocessing procedures, cross-validation strategies, hyperparameter settings, and model outputs used for evaluation are summarized in **Supplementary Table 1**.

### Model training, validation, and performance evaluation

The development cohort was randomly divided into a training set (65%) and an internal validation set (35%) using a fixed random seed (86413775). A separate hospital-based cohort was used for external comparative evaluation. Models were developed in the training set and evaluated separately in the training set, internal validation set, and external cohort. Model performance was assessed using the time-dependent area under the receiver operating characteristic curve (t-AUC), concordance index (C-index), accuracy, precision, recall, and F1-score (20). The t-AUC and C-index were calculated using continuous predicted risks or risk scores to evaluate time-dependent discrimination and overall risk ranking (21). For threshold-based metrics, including accuracy, precision, recall, and F1-score, model-specific classification thresholds were determined in the training set and then applied unchanged to the internal validation set and external cohort. Model-specific thresholds for these calculations are summarized in **Supplementary Table 2**, and definitions of all performance metrics used for model comparison are provided in **Supplementary Table 3**. The model class carried forward for further development was selected based on performance in the internal validation set and external cohort, model stability, clinical interpretability, and practical applicability.

### Extended Cox model development and validation

The proportional hazards (PH) assumption was evaluated using scaled Schoenfeld residuals and the global PH test. Restricted cubic spline (RCS) analyses assessed nonlinearity among continuous predictors retained in the multivariable Cox model (22). For the predictor showing nonproportionality, four specifications were compared: linear PH (M0), RCS under PH (M1), a linear predictor-by-log-time interaction referenced to 3 years (M2), and RCS terms with corresponding time interactions (M3); other predictors remained unchanged (23, 24). Models were compared using the Akaike information criterion (AIC), likelihood-ratio (LR) tests, performance in the internal validation set and external cohort, complexity, and parsimony (25).

In the selected model, continuous predictors were centered at training-set means. Time-specific hazard ratios (HRs) and absolute risks were estimated using Efron baseline-hazard increments and the time-dependent linear predictor (26). Three- and 4-year predictions were primary, and 5-year prediction was exploratory. Performance was evaluated using cumulative/dynamic time-dependent receiver operating characteristic (ROC) curves and corresponding AUCs, concordance indices (C-indices), Brier scores estimated using inverse probability of censoring weighting (IPCW), Kaplan-Meier observed risks, observed-to-expected (O/E) ratios, calibration curves, and decision curve analysis (DCA) (25). Harrell concordance was used for alternative-model comparisons, whereas horizon-specific C-indices were used for component and sensitivity analyses. Incremental value was assessed by comparing the FPG-only M2, non-FPG clinical, and full five-predictor M2 models in the internal validation set and external cohort without refitting or recalibration, using differences in AUC, C-index, Brier score, and absolute calibration error (27).

### Sensitivity analyses

Sensitivity analyses assessed model robustness (25, 28). Removing the post-baseline upper FPG exclusion criterion broadened the outcome to any final FPG meeting the incident-dysglycemia threshold. With predictors, functional forms, and time interaction unchanged, coefficients were re-estimated in the expanded training set and applied without recalibration to the corresponding internal validation set. Parameter stability, time-specific HRs, discrimination, prediction error, and calibration were compared with the primary analysis (28). The unchanged model was re-estimated in a cohort requiring complete data only for the final predictors, outcome status, and follow-up duration (29). Complete-case and expanded-cohort parameter sets were evaluated without recalibration in the common internal validation set and external cohort (26). Current-status interval-censoring analysis assessed event-time uncertainty. Events were represented by intervals from baseline to follow-up, whereas non-events were right-censored. Turnbull estimates were used to estimate observed risks, and a log-time interaction modeled the time-varying effect (30). Agreement with the extended Cox model was assessed using Spearman correlation coefficients for risk ranking and O/E ratios for calibration (26). This analysis was exploratory because exact event intervals were unavailable. Multivariable logistic regression adjusted for follow-up duration assessed consistency of predictor associations but was not used for survival or absolute-risk prediction. Included and excluded participants were compared to assess complete-case selection (29).

### Statistical analysis

All analyses were performed using R software (version 4.3.1). Normally distributed continuous variables were presented as mean ± standard deviation and compared using the independent-samples t test. Categorical variables were presented as number (percentage) and compared using the chi-square test. All tests were two-sided, and P < 0.05 was considered statistically significant. The development cohort was split into the training and internal validation sets, with the separate hospital-based cohort serving as the external cohort. Participants excluded during secondary screening constituted the excluded set for assessing complete-case selection. Sensitivity analyses used an expanded development cohort without the post-baseline upper-FPG exclusion and an expanded five-predictor cohort requiring complete data only for final predictors, outcome, and follow-up. Corresponding training and internal validation sets were used for model re-estimation and validation. Current-status interval-censoring analyses used the original cohorts, and the selected model structure was refitted in the full development cohort for web-based implementation.

Least absolute shrinkage and selection operator (LASSO) regression was used for variable selection and dimensionality reduction to reduce model complexity and limit overfitting (31). Variables with nonzero coefficients were incorporated into ML models and a multivariable proportional hazards Cox model. Predictors independently associated with the outcome in the initial multivariable Cox model were retained for subsequent Cox model development. HRs and 95% confidence intervals (CIs) were calculated for variables in the Cox model.

To enhance interpretability, SHapley Additive exPlanations (SHAP) analysis was applied to predictions from the final M2 model using the shapviz and survival packages in R to quantify predictor importance and illustrate predictor contributions (32, 33). SHAP feature importance and beeswarm plots were used to summarize the overall importance, direction, and magnitude of predictor effects. An interactive online risk calculator was developed using the shiny package to estimate individualized 3-, 4-, and 5-year risks of FPG-defined incident prediabetes and provide cumulative risk curves, risk stratification, and individualized SHAP-based explanations. The participant screening process and statistical workflow are shown in **Figure 1**.

**Figure 1:**
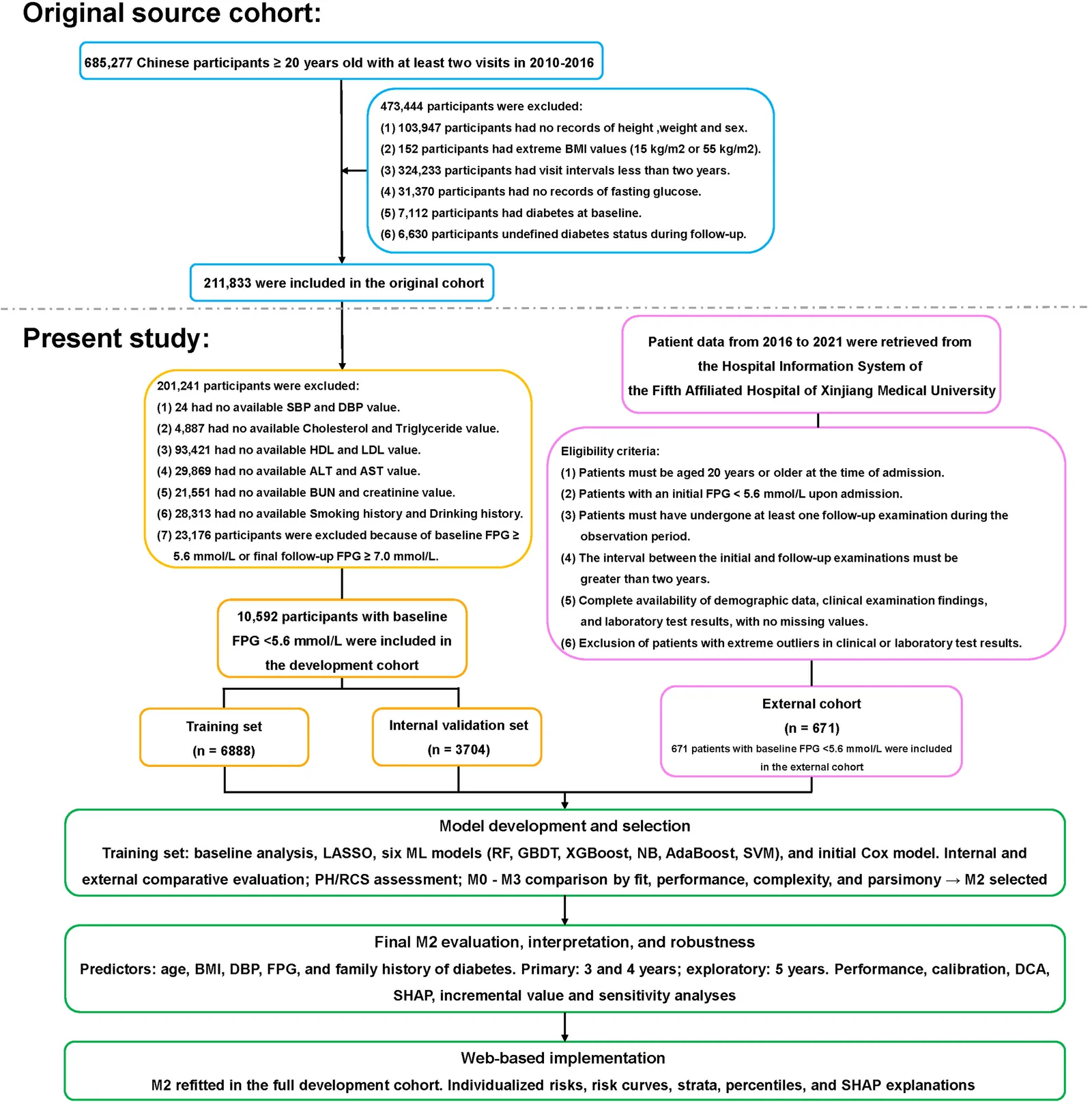
Flowchart of the study design and data analysis. BMI, body mass index; SBP, systolic blood pressure; DBP, diastolic blood pressure; HDL, high-density lipoprotein; LDL, low-density lipoprotein; ALT, alanine aminotransferase; AST, aspartate aminotransferase; BUN, blood urea nitrogen; FPG, fasting plasma glucose; LASSO, least absolute shrinkage and selection operator; ML, machine learning; RF, random forest; GBDT, gradient boosting decision tree; XGBoost, eXtreme Gradient Boosting; NB, naive Bayes; AdaBoost, adaptive boosting; SVM, support vector machine; PH, proportional hazards; RCS, restricted cubic spline; DCA, decision curve analysis; SHAP, SHapley Additive exPlanations. M0, linear FPG under the PH assumption; M1, RCS-modeled FPG under the PH assumption; M2, linear FPG-by-log-time interaction referenced to 3 years; M3, RCS-modeled FPG with corresponding time interactions.

## Results

### Study population, cohort allocation, and baseline characteristics

After data cleaning, the development cohort comprised 10, 592 eligible participants from the original database, including 6, 953 men (65.6%) and 3, 639 women (34.4%). The mean age was 40.86 ± 11.11 years, and the mean follow-up duration was 3.05 years. During follow-up, 1, 287 participants (12.2%) developed FPG-defined incident prediabetes, whereas 9, 305 participants (87.8%) did not. Baseline characteristics differed between participants with and without FPG-defined incident prediabetes (all P < 0.05; **Table 1**). Compared with participants who did not develop FPG-defined incident prediabetes, those who developed FPG-defined incident prediabetes were more frequently men, smokers, drinkers, and individuals with a family history of diabetes. They were also older and had higher BMI, SBP, DBP, FPG, cholesterol, triglycerides, LDL, ALT, AST, BUN, and creatinine levels. In contrast, HDL was lower among participants who developed FPG-defined incident prediabetes.

**Table 1:** Baseline characteristics of participants with and without FPG-defined incident prediabetes in the development set.

Variables	No FPG-defined incident prediabetes (n = 9305)	FPG-defined incident prediabetes (n = 1287)	P value
Male sex, n (%)	5992 (64.4)	961 (74.7)	< 0.01
Smoking history, n (%)	2079 (22.3)	401 (31.2)	< 0.01
Drinking history, n (%)	2031 (21.8)	336 (26.1)	< 0.01
Family history of diabetes, n (%)	513 (5.5)	117 (9.1)	< 0.01
Age, years	40.27 ± 10.9	45.11 ± 11.66	< 0.01
BMI, kg/m^2^	23.17 ± 3.20	24.65 ± 3.29	< 0.01
SBP, mmHg	117.3 ± 14.73	122.82 ± 15.39	< 0.01
DBP, mmHg	73.63 ± 10.15	77.54 ± 10.41	< 0.01
FPG, mmol/L	4.80 ± 0.48	5.04 ± 0.41	< 0.01
Cholesterol, mmol/L	4.69 ± 0.88	4.89 ± 0.88	< 0.01
Triglyceride, mmol/L	1.34 ± 1.03	1.69 ± 1.09	< 0.01
HDL, mmol/L	1.36 ± 0.3	1.28 ± 0.29	< 0.01
LDL, mmol/L	2.69 ± 0.68	2.83 ± 0.68	< 0.01
ALT, U/L	24.52 ± 20.86	30.19 ± 26.78	< 0.01
AST, U/L	24.42 ± 10.13	26.18 ± 12.33	< 0.01
BUN, mmol/L	4.67 ± 1.16	4.88 ± 1.18	< 0.01
Creatinine, μmol/L	73.16 ± 15.32	74.93 ± 14.59	< 0.01

Data are shown as means ± standard deviation for normally distributed variables and as percentages for categorical variables. BMI, body mass index; SBP, systolic blood pressure; DBP, diastolic blood pressure; FPG, fasting plasma glucose; HDL, high-density lipoprotein; LDL, low-density lipoprotein; ALT, alanine aminotransferase; AST, aspartate aminotransferase; BUN, blood urea nitrogen.

Using a fixed random seed, the development cohort was randomly divided into a training set (n=6, 888) and an internal validation set (n=3, 704) at a ratio of 6.5:3.5. Baseline characteristics were well balanced between the training and internal validation sets, with no statistically significant differences in measured baseline variables (all P > 0.05; **Supplementary Table 4**). In the training set, participants who developed FPG-defined incident prediabetes showed a baseline risk profile broadly consistent with that observed in the overall development cohort (all P < 0.05; **Supplementary Table 5**).

A separate hospital-based cohort from the Fifth Affiliated Hospital of Xinjiang Medical University included 671 participants, with a mean follow-up duration of 3.02 years. Among them, 75 participants (11.2%) developed FPG-defined incident prediabetes and 596 participants (88.8%) did not. Measured baseline characteristics did not differ significantly between the development cohort and the external cohort (all P > 0.05; **Supplementary Table 6**). Within the external cohort, participants who developed FPG-defined incident prediabetes were older and had higher BMI, SBP, DBP, FPG, triglycerides, ALT, and AST levels, as well as a higher prevalence of family history of diabetes, than those who did not develop FPG-defined incident prediabetes. HDL was slightly lower in participants who developed FPG-defined incident prediabetes, although this difference was not statistically significant (**Supplementary Table 7**).

### LASSO-based predictor selection and comparative model performance

To identify candidate predictors for model development, categorical variables were numerically encoded, and LASSO regression was applied to the 17 candidate clinical variables in the training set. Based on 10-fold cross-validation, the optimal penalty parameter was lambda.min = 0.002839. This value yielded the lowest prediction error and thus achieved an optimal balance between retaining potential predictive information and improving model fit. Ultimately, 11 variables with nonzero coefficients were selected: age, BMI, DBP, FPG, triglycerides, ALT, BUN, sex, smoking history, drinking history, and family history of diabetes (**Figure 2**; **Supplementary Table 8**).

**Figure 2:**
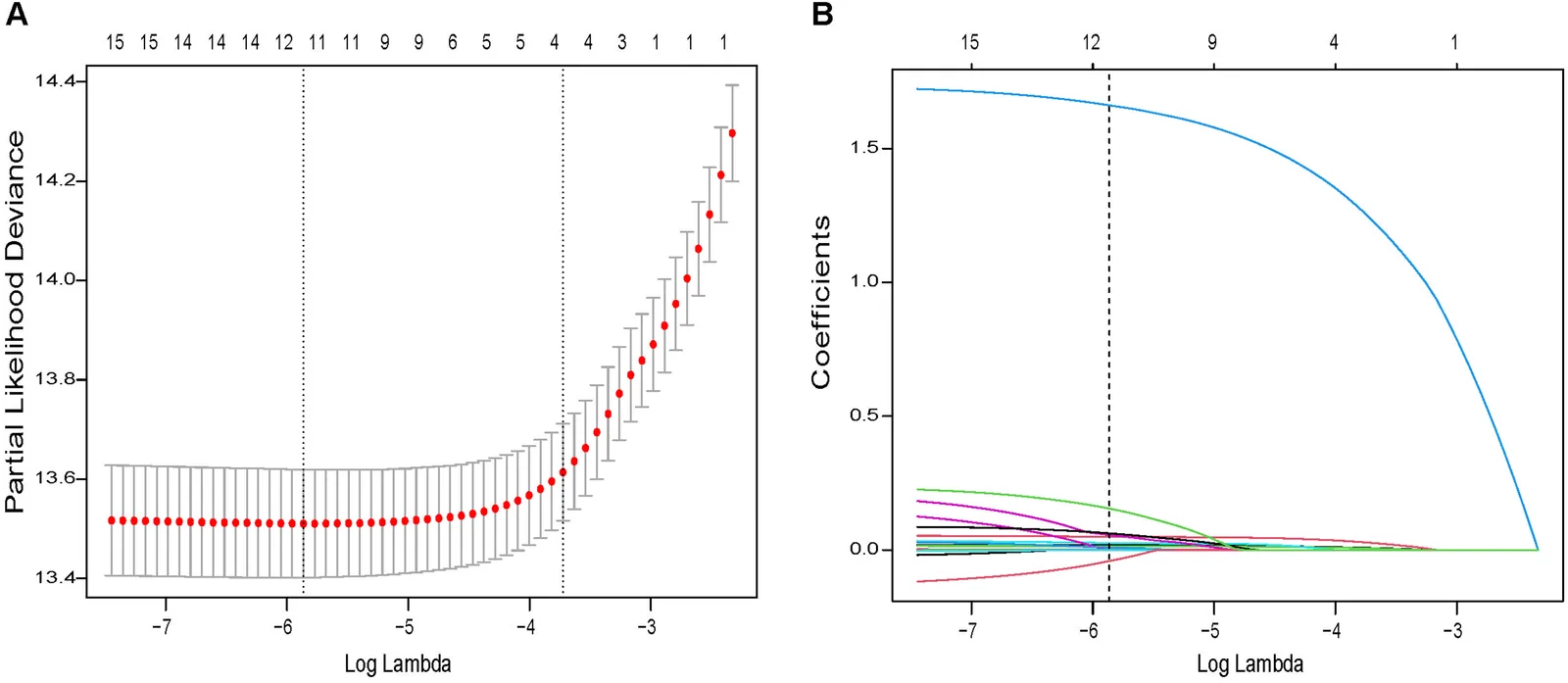
Least absolute shrinkage and selection operator-based predictor selection in the training set. **(A)** Cross-validation curve for selection of the optimal penalty parameter. **(B)** Coefficient profiles of candidate predictors. LASSO, least absolute shrinkage and selection operator.

Among the models evaluated in the training set, RF showed the highest apparent performance, with a time-dependent AUC (t-AUC) of 0.936 and a C-index of 0.936 (95% CI, 0.931-0.942). Its accuracy, precision, recall, and F1-score were 0.979, 0.984, 0.993, and 0.975, respectively. However, the performance of RF decreased substantially in the internal validation set and external cohort, in which its t-AUCs were 0.717 and 0.685 and its C-indices were 0.704 and 0.675, respectively. The other machine-learning models also showed varying degrees of reduction in predictive performance across the evaluation datasets (**Figure 3**; **Supplementary Table 9**).

**Figure 3:**
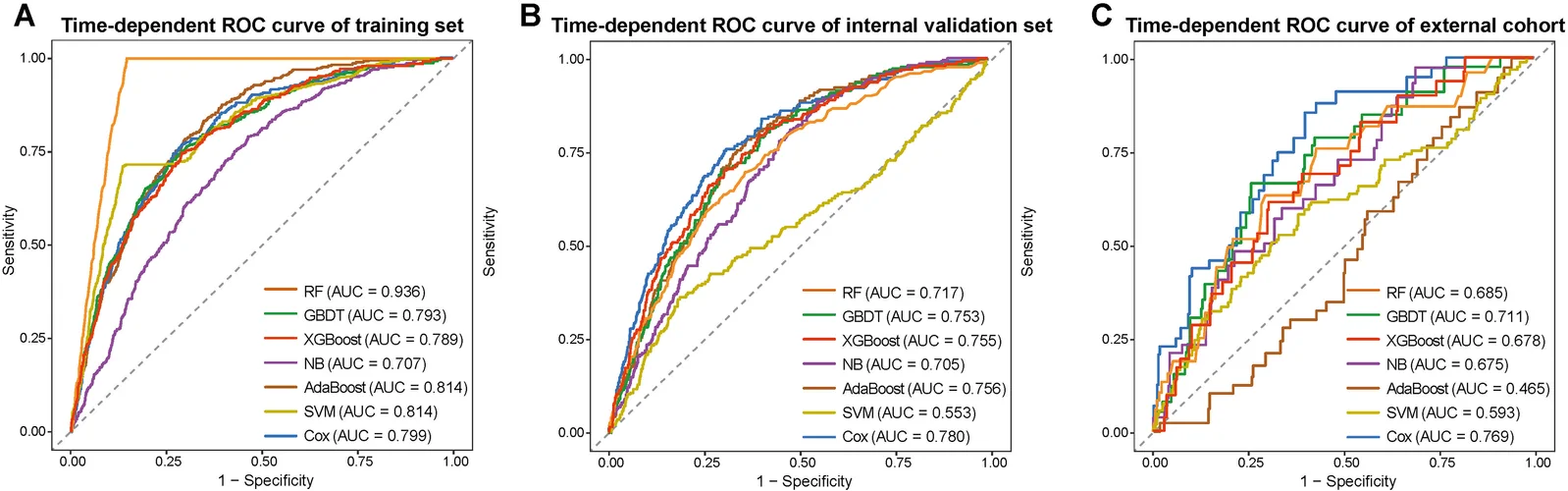
Time-dependent receiver operating characteristic curves of six machine-learning models and the initial proportional-hazards Cox model. **(A)** Based on the training set. **(B)** Based on the internal validation set. **(C)** Based on the external cohort. ROC, receiver operating characteristic; AUC, area under the curve; FPG, fasting plasma glucose; RF, random forest; GBDT, gradient boosting decision tree; XGBoost, eXtreme Gradient Boosting; NB, naive Bayes; AdaBoost, adaptive boosting; SVM, support vector machine; Cox, proportional hazards Cox model.

In the internal validation set, the initial proportional-hazards Cox model showed the highest t-AUC of 0.780 and the highest C-index of 0.756 (95% CI, 0.732-0.781), with a recall of 0.777. By comparison, the t-AUCs of the GBDT, XGBoost, and AdaBoost models ranged from 0.753 to 0.756, and their C-indices ranged from 0.727 to 0.732. In the external cohort, the initial Cox model retained the highest t-AUC of 0.769 and the highest C-index of 0.734 (95% CI, 0.660-0.796), together with a recall of 0.882. Among the machine-learning models, GBDT showed the highest t-AUC in the external cohort of 0.711 and C-index of 0.681 (**Figure 3**). Overall, although RF showed strong apparent performance in the training set, the initial Cox model demonstrated the most consistent discrimination across the internal validation set and external cohort.

### Assessment of proportional hazards and selection of the final extended Cox model

In the initial multivariable proportional-hazards Cox model, age, BMI, DBP, FPG, and family history of diabetes were significantly associated with FPG-defined incident prediabetes. Among these predictors, baseline FPG showed the strongest association with the outcome (HR, 5.65; 95% CI, 4.71-6.78), whereas age, BMI, DBP, and family history of diabetes showed more modest positive associations. Triglycerides, ALT, BUN, sex, smoking history, and drinking history were not statistically significant in the multivariable analysis (**Supplementary Table 10**).

Assessment of Schoenfeld residuals indicated that the proportional-hazards assumption was violated for FPG and for the model globally (both P < 0.05), whereas no statistically significant violations were detected for the remaining predictors (**Supplementary Table 11**). Restricted cubic spline analyses showed significant overall associations of age, BMI, DBP, and FPG with the outcome. Evidence of nonlinearity was identified for FPG (P for nonlinearity = 0.013), but not for age, BMI, or DBP, supporting further investigation of both the functional form and time-varying effect of FPG (**Supplementary Figure 1**).

Accordingly, four alternative Cox models were systematically compared. Modeling FPG using restricted cubic splines under proportional hazards (M1) improved model fit relative to the linear proportional-hazards model (M0). However, the extended Cox model incorporating a linear FPG-by-log-time interaction (M2) provided the best overall fit, with the lowest AIC and a significant improvement over M0 (P < 0.001). Adding nonlinear spline terms and their corresponding time interactions in M3 did not provide a significant improvement over M2 (P = 0.318), indicating that the more complex parameterization was not supported by the data (**Table 2**).

**Table 2:** Comparison of alternative functional forms and time-varying effects for baseline fasting plasma glucose in the training set.

Model	FPG specification	No. of parameters	AIC	Delta AIC	LR χ² (df), comparison	P value
M0	Linear FPG; proportional hazards	5	12, 464.289	18.498	Reference	–
M1	Restricted cubic spline FPG; proportional hazards	6	12, 459.746	13.955	6.543 (1)	0.011
M2	Linear FPG with FPG × log(t/3)	6	12, 445.790	0.000	20.498 (1)	<0.001
M3	Restricted cubic spline FPG with corresponding time interactions	8	12, 447.501	1.710	2.290 (2), vs M2	0.318

Likelihood-ratio tests compared M1 and M2 with M0 and compared M3 with M2. ΔAIC values were calculated relative to the model with the lowest AIC using unrounded estimates; lower AIC values indicate better relative model fit. M0, linear FPG under proportional hazards; M1, restricted cubic spline FPG under proportional hazards; M2, linear FPG with FPG × log(t/3); M3, restricted cubic spline FPG with corresponding time interactions; FPG, fasting plasma glucose; AIC, Akaike information criterion; LR, likelihood ratio; df, degrees of freedom.

The four models showed generally similar discrimination and calibration across the internal validation set and external cohort, with no consistent validation advantage observed for the more complex M3 model over M2. M2 also showed Brier scores and O/E ratios comparable to, or slightly better than, those of the alternative models at the evaluated prediction horizons. Performance at the 5-year horizon was interpreted cautiously because only 75 and 20 participants remained at risk in the internal and external cohort, respectively (**Supplementary Table 12**). Considering its accommodation of the time-varying FPG effect, superior model fit, comparable performance across the internal validation set and external cohort, and greater parsimony relative to M3, M2 was selected as the final extended Cox model.

In the final M2 model, age, BMI, and DBP remained positively associated with FPG-defined incident prediabetes. Family history of diabetes showed a positive association, although its confidence interval included the null value. At the 3-year reference time, each 1-mmol/L increase in baseline FPG was associated with a 6.33-fold higher hazard (95% CI, 5.23-7.67). The negative FPG-by-log-time interaction coefficient (β = -1.493, P < 0.001) confirmed that the association between baseline FPG and the outcome weakened over time (**Supplementary Table 13**). Specifically, the adjusted HR per 1-mmol/L higher baseline FPG decreased from 11.60 at 2 years to 6.33 at 3 years, 4.12 at 4 years, and 2.95 at 5 years, while remaining above the null throughout the evaluated period (**Supplementary Figure 2**).

The baseline cumulative hazard, baseline survival, and reference-profile risk estimates required for 3-, 4-, and 5-year absolute-risk prediction are provided in **Supplementary Table 14**. Exact individual risk estimates were calculated using the locked Efron baseline-hazard increments from all 429 distinct event times in the training set rather than a conventional fixed-linear-predictor Cox formula.

### Discrimination, calibration, and clinical net benefit of the final M2 model

The final M2 model maintained consistent discrimination at the primary 3- and 4-year horizons across the three cohorts. The respective AUCs were 0.800 and 0.796 in the training set, 0.778 and 0.755 in the internal validation set, and 0.750 and 0.764 in the external cohort. At the 5-year horizon, the AUCs were 0.761, 0.739, and 0.667, respectively (**Figure 4**). Calibration curves generally followed the reference line, particularly at the primary 3- and 4-year horizons. Greater deviations and wider uncertainty intervals were observed at higher predicted-risk levels and for 5-year predictions in the validation sets, consistent with the smaller effective sample sizes at longer follow-up durations (**Supplementary Figure 3**). DCA indicated that M2 provided greater net benefit than the treat-all and treat-none strategies across a range of threshold probabilities for 3- and 4-year prediction in all three cohorts. The 5-year model also showed positive net benefit in the training and internal validation sets, whereas its curve for the external cohort was less stable at higher thresholds. These findings indicate favorable decision-analytic performance of M2 for individualized 3- and 4-year risk estimation across the evaluated threshold-probability ranges and support its further evaluation in clinical settings; however, they do not establish clinical effectiveness. Five-year predictions were considered exploratory because of their reduced stability in the external cohort (**Supplementary Figure 4**).

**Figure 4:**
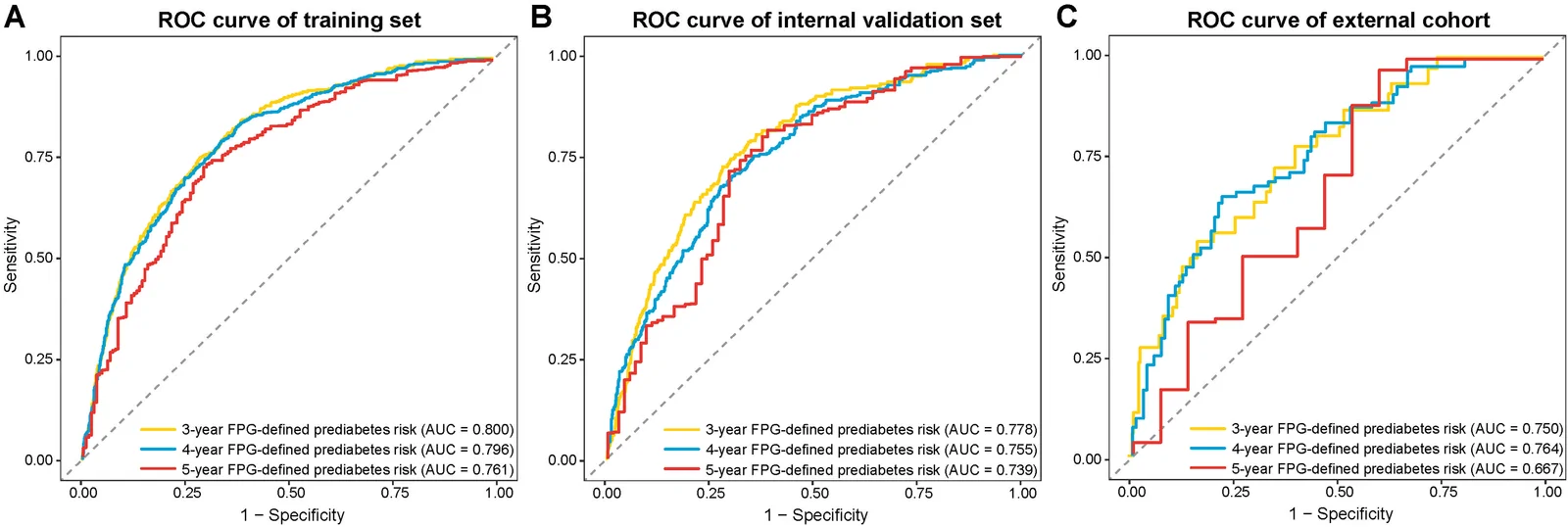
Receiver operating characteristic curves for 3-, 4-, and 5-year prediction using the final extended Cox model (M2). **(A)** Based on the training set. **(B)** Based on the internal validation set. **(C)** Based on the external cohort. ROC, receiver operating characteristic; AUC, area under the ROC curve; FPG, fasting plasma glucose; M2, the final extended Cox model incorporating FPG × log(t/3).

### SHAP-based interpretation and incremental predictive value of the final M2 model

SHAP-based interpretation of the final M2 model identified FPG as the dominant contributor to individual risk predictions, followed by BMI, age, DBP, and family history of diabetes. The beeswarm plot showed that higher FPG values were consistently associated with higher predicted log-risk. Higher BMI, age, and DBP values also generally contributed to increased predicted risk, whereas family history of diabetes had a comparatively modest contribution (**Figure 5**). Comparisons of the component models further supported the central contribution of FPG to prediction. At the primary 3- and 4-year horizons, the full five-predictor M2 model showed substantially better discrimination than the non-FPG clinical model in both validation cohorts. By contrast, its performance was closer to that of the FPG-only M2 model, indicating that FPG accounted for a large proportion of the model’s discriminative information. Nevertheless, adding age, BMI, DBP, and family history of diabetes provided modest improvements in concordance and calibration, particularly in the internal validation set (**Supplementary Table 15**). Compared with the FPG-only M2 model, the full model increased the AUC by 0.018 and 0.013 and the C-index by 0.022 and 0.019 at 3 and 4 years, respectively, in the internal validation set. The corresponding changes in discrimination in the external cohort were small, with changes in AUC ranging from −0.001 to 0.002 and changes in the C-index from 0.001 to 0.002. However, the full model generally reduced calibration error and showed small improvements in Brier scores, particularly at the 4-year horizon and in the external cohort. Incremental estimates at 5 years were less consistent and were therefore interpreted cautiously (**Supplementary Table 16**). Overall, FPG provided most of the discriminatory information, whereas the additional clinical predictors offered modest complementary value, primarily through improved internal discrimination and calibration.

**Figure 5:**
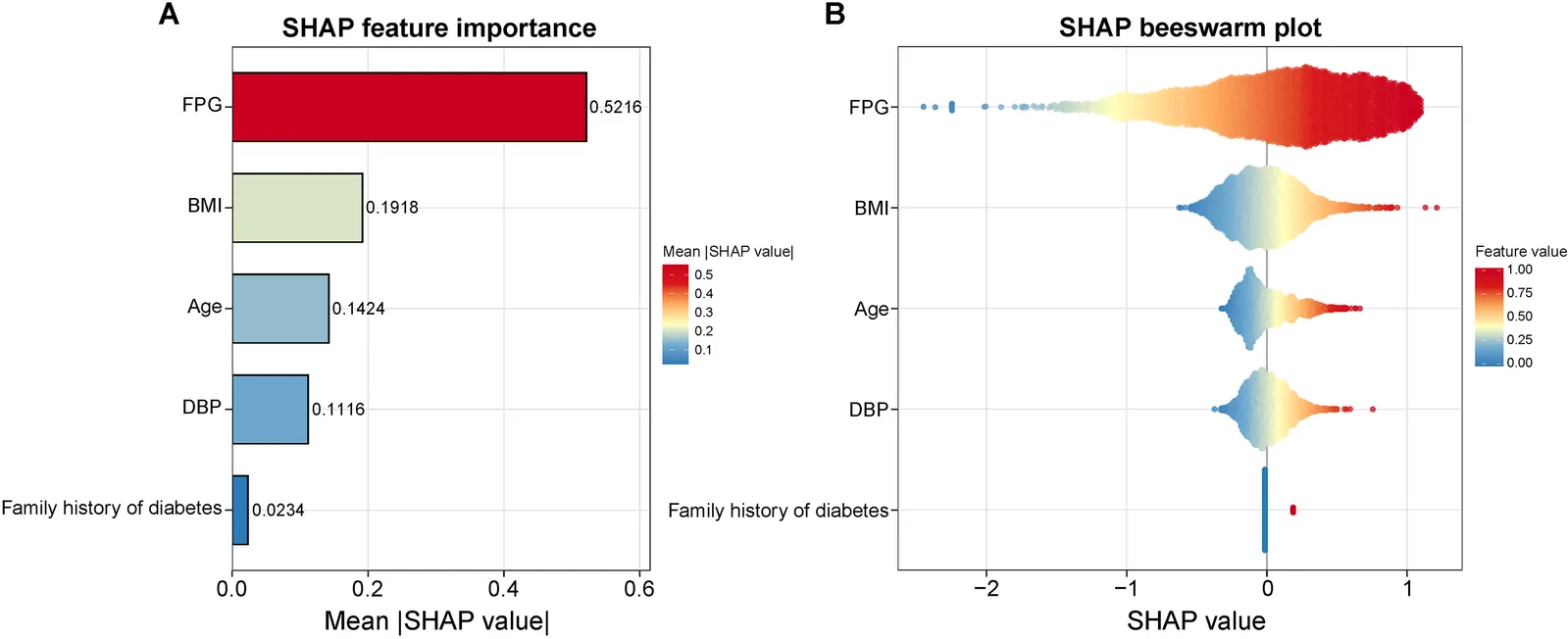
SHapley Additive exPlanations-based interpretation of the final extended Cox model (M2). **(A)** SHAP feature importance. **(B)** SHAP beeswarm plot. SHAP, SHapley Additive exPlanations; BMI, body mass index; DBP, diastolic blood pressure; FPG, fasting plasma glucose.

### Sensitivity analyses and robustness of the final M2 model

Removal of the post-baseline upper-FPG exclusion criterion reintroduced 50 participants with outcome events, increasing the development cohort to 10, 642 participants. After refitting the prespecified M2 structure, relative changes in the regression coefficients and time-specific FPG HRs were below 6%, and internal-validation discrimination, prediction error, and calibration remained similar to those in the primary analysis. The direction and time-varying pattern of the FPG association were therefore unaffected by this exclusion criterion (**Supplementary Tables 17–19**).

M2 was subsequently re-estimated in an expanded cohort of 185, 563 participants with complete data for the five final predictors. All predictor effects retained their original directions, although the magnitude of the FPG association was attenuated. The FPG HR nevertheless continued to decline over time, from 7.44 at 2 years to 2.79 at 5 years. Evaluation in common validation populations showed nearly identical discrimination between the complete-case and expanded-cohort parameter sets. Re-estimation in the expanded cohort improved calibration in the expanded internal validation set and at the primary 3- and 4-year prediction horizons in the external cohort, although 5-year calibration in the external cohort remained less stable (**Supplementary Tables 20**-**22**).

The current-status interval-censoring analysis yielded highly concordant individual risk rankings with M2 across cohorts and prediction horizons (Spearman ρ = 0.951–0.974) and reproduced the declining FPG association over time. However, the interval-censoring model produced smaller and less precise FPG effect estimates at later horizons, and its absolute-risk estimates differed from those of M2, particularly at 4 and 5 years. Thus, risk ranking and the direction of the time-varying association were robust, whereas absolute calibration was more sensitive to assumptions regarding event timing (**Supplementary Tables 23**, **24**).

In a complementary logistic regression analysis adjusted for follow-up duration, all five predictors remained positively associated with FPG-defined incident prediabetes, with FPG showing the strongest association (adjusted OR, 4.31; 95% CI, 3.52–5.31). Longer follow-up was also associated with a higher probability of outcome detection, but this auxiliary model was not used for absolute-risk prediction (**Supplementary Table 25**). The sensitivity analyses supported the robustness of the predictor directions, the declining FPG effect over time, and the discriminatory performance of M2.

### Web-based implementation of the final M2 model for individualized risk prediction

To facilitate individualized risk estimation, an interactive web-based risk calculator was developed using the Shiny package in R based on the selected M2 model structure refitted in the full development cohort. The calculator incorporates age, BMI, DBP, FPG, and family history of diabetes to estimate individualized 3-, 4-, and 5-year risks of FPG-defined incident prediabetes and provides cumulative risk curves, risk stratification, risk percentiles, and individualized SHAP-based explanations. In the representative example, a 38-year-old individual with a BMI of 23.2 kg/m², DBP of 73 mmHg, FPG of 4.9 mmol/L, and no family history of diabetes was classified as being at intermediate risk, with predicted 3-, 4-, and 5-year risks of 5.5%, 16.0%, and 41.9%, respectively, corresponding to the 47th percentile of the development-cohort risk distribution. The individualized SHAP waterfall plot showed that age, BMI, FPG, and the absence of a family history of diabetes shifted the prediction toward lower risk, whereas DBP made a small contribution toward higher risk (**Figure 6**). These results suggest that the calculator may support individualized risk estimation and interpretable risk stratification for FPG-defined incident prediabetes, although further independent external validation is needed before routine clinical use.

**Figure 6:**
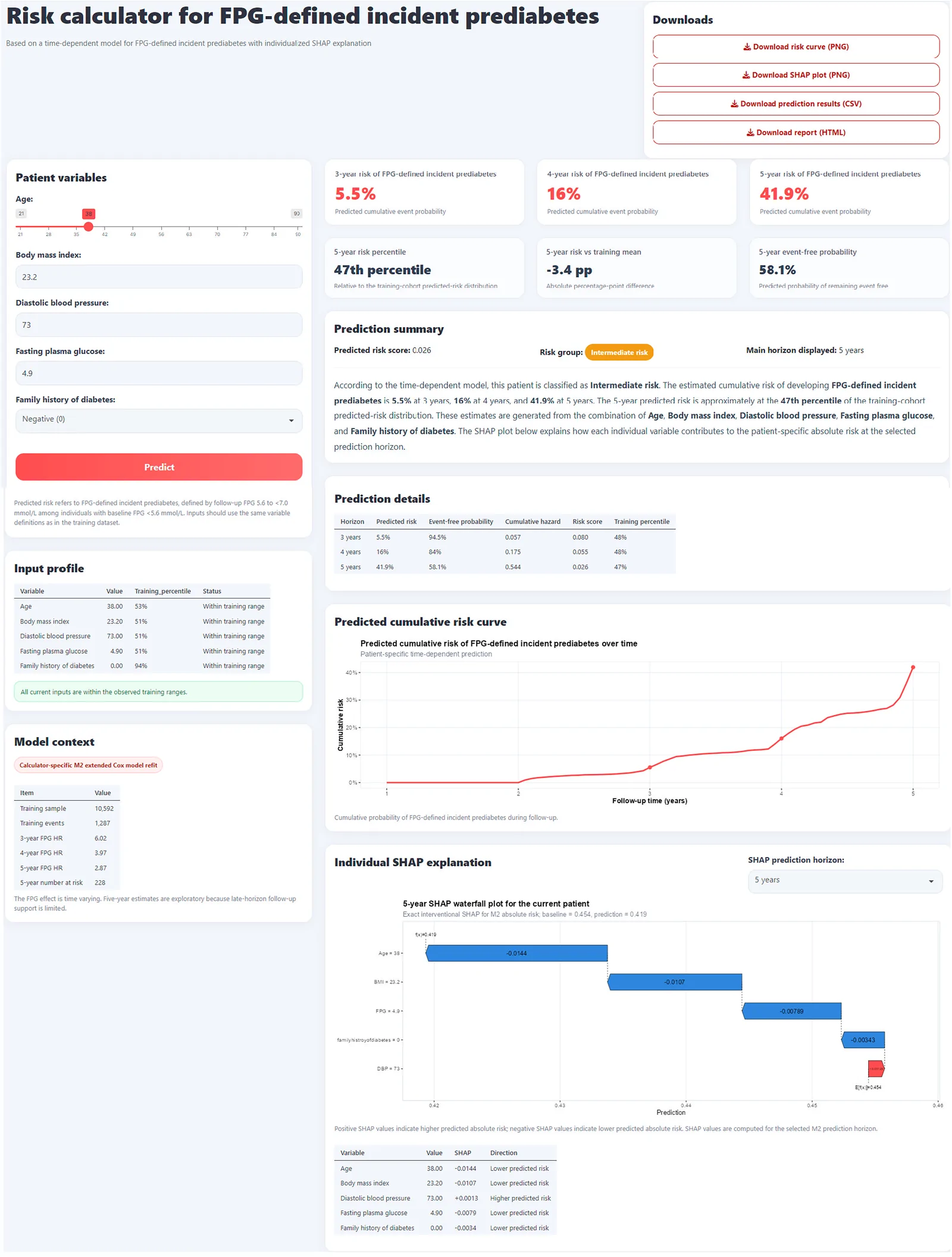
Web-based implementation of the final extended Cox model (M2) for individualized prediction of FPG-defined incident prediabetes. The calculator provides individualized 3-, 4-, and 5-year risk estimates derived from the final M2 model and is available at: https://019d4d4f-5277-5cd7-c69c-2914663f46ed.share.connect.posit.cloud/. SHAP, SHapley Additive exPlanations; BMI, body mass index; DBP, diastolic blood pressure; FPG, fasting plasma glucose; M2, the final extended Cox model incorporating FPG × log(t/3).

## Discussion

Diabetes is a major global public health challenge because of its chronic course, high disease burden, and close association with cardiovascular disease, kidney disease, retinopathy, neuropathy, and premature mortality (34). China bears a particularly substantial burden of diabetes, driven by rapid socioeconomic transition, population aging, increasing adiposity, urbanization, and lifestyle changes, including unhealthy dietary patterns and reduced physical activity (35). Because diabetes usually develops gradually and may remain clinically silent for years, identifying individuals at an earlier stage of dysglycemia is important for prevention and risk management (2). Prediabetes represents an intermediate metabolic state associated with increased risk of diabetes, cardiovascular disease, and other adverse outcomes (36). However, in large-scale health examination cohorts, hemoglobin A1c (HbA1c) and oral glucose tolerance test (OGTT) data are often unavailable, making FPG a practical and widely available marker for early glycemic risk assessment. Therefore, prediction models targeting recorded FPG-defined incident prediabetes may provide a structured approach to estimating future glycemic risk among adults with normal fasting glucose. In this context, the present study compared ML and Cox regression approaches, accounted for the time-varying association between baseline FPG and the outcome, and developed an interpretable extended Cox model (M2) based on five routinely available predictors. The final model was presented through an online risk calculator and evaluated in a separate hospital-based cohort, providing external comparative evidence regarding its predictive performance and potential transportability for individualized 3- and 4-year risk estimation.

In this cohort study of 10, 592 Chinese adults with normal fasting glucose at baseline, 1, 287 participants (12.2%) developed FPG-defined incident prediabetes during a mean follow-up of 3.05 years. We developed and compared six ML models and an initial proportional-hazards Cox model, with performance assessed in the training set, internal validation set, and external cohort. LASSO regression retained 11 of 17 candidate baseline predictors for model development. Although RF showed the highest apparent performance in the training set, its performance declined in the internal validation set and external cohort, suggesting overfitting and limited transportability. In contrast, the initial Cox model demonstrated more consistent discrimination across the evaluation datasets while retaining greater interpretability and clinical simplicity. Diagnostic assessment subsequently identified a nonproportional association between FPG and incident prediabetes, prompting a systematic comparison of four Cox specifications. The extended Cox model incorporating FPG × log(t/3) (M2) had the lowest Akaike information criterion, accommodated the attenuation of the FPG association over time, and achieved predictive performance comparable to that of more complex alternatives; it was therefore selected as the final model. M2 retained age, BMI, DBP, FPG, and family history of diabetes as predictors. At the primary 3- and 4-year horizons, the model showed generally reasonable discrimination, calibration, and clinical net benefit, including 3- and 4-year AUCs of 0.750 and 0.764, respectively, in the external cohort. Five-year performance was exploratory and less stable in the external cohort. These findings support the potential value of the final M2 model and online risk calculator as an interpretable framework for individualized 3- and 4-year risk estimation of recorded FPG-defined incident prediabetes. Five-year prediction should be considered exploratory, and truly independent external validation, recalibration where necessary, and prospective clinical impact evaluation are required before clinical implementation.

Among the five predictors retained in M2, age is an established and nonmodifiable risk factor for diabetes development. As people grow older, especially in the elderly, the function of pancreatic islet cells declines to varying degrees, leading to insufficient insulin secretion and increased risk of diabetes (2, 4). Additionally, the probability of prediabetes in adolescents is also significantly increased, and there is a greater chance of developing diabetes. Moreover, early-onset vascular complications and other complications related to long-term exposure to abnormal plasma glucose are more concealed and have a more significant impact. This is because adolescents are more susceptible to the effects of unhealthy lifestyles and obesity, with more pronounced insulin resistance (IR) and significant damage to β-cell function at an early stage (37). Therefore, it is necessary to raise awareness of prediabetes across all age groups. Although age is an uncontrollable factor, interventions in lifestyle habits, such as a healthy diet and increased physical activity, can protect β-cell function and improve IR.

Generally, BMI is the most intuitive indicator of body fat burden. Individuals who are overweight or obese often experience IR, which means that body tissues (such as muscles, fat, and liver) become less sensitive to insulin. This significantly reduces the efficiency of insulin in promoting glucose uptake, utilization, and storage. As a result, the body needs to produce more insulin to meet its demands, and the inability of pancreatic β-cells to compensate effectively accelerates the onset of diabetes (38, 39). A nationwide cohort study in China found that BMI is positively associated with prediabetes in a dose-response manner. For Chinese people with normal weight, attention should be paid to glucose metabolism when BMI exceeds 23.03 kg/m² (40). As a modifiable risk factor for prediabetes, BMI can be effectively managed through a healthy diet and exercise, especially for individuals with abdominal obesity. Setting BMI as a target can effectively reduce the chronic inflammatory response and IR caused by obesity, thereby actively controlling plasma glucose levels (39, 41). However, the interpretation of BMI should be done cautiously on an individual basis. For example, athletes or people with a long-term exercise habit may have a higher BMI due to increased muscle mass, and this should not be generalized.

DBP is a key indicator of peripheral vascular resistance and can reflect endothelial function non-invasively. Even when it does not meet the criteria for hypertension, elevated DBP is significantly associated with metabolic abnormalities and drives the development of persistent metabolic disorders through mechanisms such as inflammation and vascular dysfunction (42). Pan et al. (43) retrospectively analyzed 183, 517 adults aged over 20 years from the Fu De Health Group in China between 2010 and 2016. After a 3-year follow-up, they found that DBP is an independent risk factor for prediabetes. For every 1-mmHg increase in DBP, the risk of prediabetes increased by 3% (HR, 1.030, 95% CI, 1.029-1.031, P < 0.001), which is consistent with our findings. For individuals with elevated DBP alone, regular screening of glucose metabolism indicators and attention to plasma glucose health are necessary.

Approximately 10% of individuals with prediabetes in the United States progress to type 2 diabetes mellitus annually, and the risk of diabetes increases with higher FPG levels (2). Consistent with this evidence, baseline FPG showed a pronounced time-varying association with FPG-defined incident prediabetes in the final extended Cox model, although the magnitude of this association declined over time. FPG also emerged as the most influential predictor in the SHAP analysis, consistent with its central contribution to model-based risk estimation. Because the outcome was itself defined using a future FPG threshold, this dominant contribution may partly reflect a threshold-proximity effect and should not, by itself, be interpreted as evidence of established clinical utility. Beyond FPG, family history of diabetes was retained as a predictor in M2, consistent with previous evidence that individuals with a family history of diabetes are more likely to exhibit insulin resistance, impaired insulin secretion, and metabolic syndrome (44).

Compared with the relative opacity of many ML models, the final extended Cox model (M2) provides a more transparent and clinically interpretable framework while explicitly accommodating the time-varying association between FPG and incident prediabetes (45). SHAP analyses complemented the model coefficients by characterizing the relative contribution and direction of each predictor at both the population and individual levels, thereby improving the interpretability of the model and its risk estimates. The five final predictors identified in this study captured complementary dimensions of risk for FPG-defined incident prediabetes. Among them, BMI and DBP are modifiable clinical indicators that may be improved through lifestyle modification and blood pressure management, whereas FPG serves as a practical marker of early glycemic risk. Age and family history of diabetes are nonmodifiable but remain useful for identifying individuals with higher underlying susceptibility. Because all five predictors are routinely available in clinical and health-examination settings, the model may be feasible for further external validation and future implementation studies. The routine availability of these predictors may also facilitate their integration into digital risk-assessment platforms (46). Our online risk calculator translates the final M2 model into an accessible interface that presents individualized risk estimates, development-cohort-based risk categories and percentiles, and predictor-specific explanations. The 3- and 4-year estimates represent the primary prediction horizons, whereas the 5-year estimates should be interpreted cautiously because of their exploratory nature and reduced stability in the external cohort. Collectively, the model and calculator provide an interpretable and potentially practical framework for individualized risk estimation and risk communication. However, truly independent external validation, setting-specific recalibration, and prospective clinical impact evaluation are required before routine clinical use.

This study has several limitations. First, the outcome was restricted to FPG-defined incident prediabetes because HbA1c and OGTT data were unavailable. Individuals with isolated elevated HbA1c or IGT were therefore not captured, limiting generalization to broader definitions of prediabetes. Nevertheless, applying a uniform FPG threshold across cohorts provided a clearly defined and clinically accessible endpoint. Repeated confirmatory FPG measurements were not consistently available; however, uniform criteria were used, and participants with identifiable glucose-lowering medication use or acute conditions affecting glucose metabolism were excluded from the external cohort. Second, complete visit-level FPG trajectories and exact dates of the last normal and first abnormal examinations were unavailable; therefore, M2 predicts time to recorded detection rather than biological onset. The exploratory current-status interval-censoring analysis reproduced the declining FPG association and showed highly concordant risk rankings with M2 (Spearman ρ = 0.951-0.974), although absolute-risk estimates remained sensitive to event-time assumptions. Third, complete-case selection may have introduced bias. We compared included and excluded participants to characterize potential selection differences (**Supplementary Table 26**). More importantly, re-estimating M2 in 185, 563 participants with complete data for the five final predictors preserved predictor directions and the time-varying FPG pattern, produced nearly identical discrimination, and improved 3- and 4-year calibration, supporting the robustness of the main findings. Fourth, the parsimonious model prioritized five routinely available indicators, but more detailed anthropometric, lifestyle, medication, and longitudinal risk-factor data were unavailable and warrant future evaluation. Finally, because external-cohort performance informed model comparison and selection, this cohort was not reserved exclusively for independent external validation, potentially introducing validation bias and model-selection-related optimism. Nevertheless, it provided external comparative evidence from a separate hospital setting. Given its retrospective, single-center nature, independent multicenter external validation, recalibration where necessary, and prospective clinical impact studies are required before routine implementation.

## Conclusion

We developed and evaluated an interpretable extended Cox model (M2) incorporating age, BMI, DBP, FPG, and family history of diabetes to estimate the 3- and 4-year risk of recorded detection of FPG-defined incident prediabetes among Chinese adults with normal fasting glucose. By accommodating the time-varying association between baseline FPG and the outcome, M2 demonstrated moderate discrimination, together with generally favorable calibration and decision-analytic performance at the primary prediction horizons, and showed a favorable balance of model fit, stability, interpretability, and parsimony relative to the alternative models assessed. Five-year predictions were less stable, particularly in the external cohort, and should be considered exploratory. Together with SHAP-based interpretation and the online risk calculator, M2 provides an interpretable and potentially practical framework for individualized risk estimation using routinely available predictors. Because the external cohort contributed to model comparison and selection, truly independent multicenter external validation, recalibration where necessary, and prospective evaluation of clinical impact are required before clinical implementation.

## Data Availability

The original contributions presented in the study are included in the article/**Supplementary Material**. Further inquiries can be directed to the corresponding author.
