# Supplementary material for: Development and comparative evaluation of machine learning algorithms and Cox regression for predicting fasting plasma glucose-defined incident prediabetes: a longitudinal cohort study
Source: Front Endocrinol (Lausanne). 2026 Sep 24;17:1862258. doi: 10.3389/fendo.2026.1862258 (PMC13645560; doi:10.3389/fendo.2026.1862258)
Supplement: Supplementary file 1 [file Table1.docx]

**Supplementary Table 1. Hyperparameter search space and final selected parameters for machine learning models.**

| Model | Modeling framework | Data preprocessing | Cross-validation and selection criterion | Hyperparameter search space or fixed settings | Final parameter setting | Model output used for evaluation |
| --- | --- | --- | --- | --- | --- | --- |
| Random forest | Classification-based risk score model | Original predictors; categorical variables encoded as factors | 5-fold cross-validation; mean ROC/AUC used for model selection | mtry = 2, 3, 4, 5; ntree = 500 | mtry = 3; ntree = 500 | Predicted probability of Event_Yes |
| Gradient boosting decision tree | Classification-based risk score model | Original predictors; categorical variables encoded as factors | 5-fold cross-validation; mean ROC/AUC used for model selection | n.trees = 50, 100, 150, 200; interaction.depth = 1, 2, 3; shrinkage = 0.01, 0.05, 0.10; n.minobsinnode = 5, 10 | n.trees = 100; interaction.depth = 3; shrinkage = 0.1; n.minobsinnode = 5 | Predicted probability of Event_Yes |
| XGBoost | Classification-based risk score model | Dummy encoding of categorical predictors | Manual 5-fold cross-validation with out-of-fold predicted probabilities | nrounds = 100; max_depth = 3; learning_rate = 0.10; min_split_loss = 0; colsample_bytree = 0.80; min_child_weight = 1; subsample = 0.80 | nrounds = 100; max_depth = 3; learning_rate = 0.10; min_split_loss = 0; colsample_bytree = 0.80; min_child_weight = 1; subsample = 0.80 | Predicted probability of Event_Yes |
| Naive Bayes | Classification-based risk score model | Original predictors; categorical variables encoded as factors | 5-fold cross-validation | laplace = 1; usekernel = FALSE; adjust = 1 | laplace = 1; usekernel = FALSE; adjust = 1 | Predicted probability of Event_Yes |
| AdaBoost | Classification-based risk score model | Original predictors; categorical variables encoded as factors | Manual 5-fold cross-validation with out-of-fold predicted probabilities | mfinal = 100; maxdepth = 2; coeflearn = Zhu | mfinal = 100; maxdepth = 2; coeflearn = Zhu | Predicted probability of Event_Yes |
| Support vector machine | Classification-based risk score model with radial kernel | Dummy encoding followed by centering and scaling | Repeated 5-fold cross-validation with 3 repeats; mean ROC/AUC used for model selection | sigma = 0.001, 0.01, 0.10; C = 0.1, 1, 10 | sigma = 0.1; C = 1 | Predicted probability of Event_Yes |

XGBoost, eXtreme gradient boosting; AdaBoost, adaptive boosting; ROC, receiver operating characteristic curve; AUC, area under the curve; FPG, fasting plasma glucose.

**Supplementary Table 2. Classification thresholds used for the calculation of accuracy, precision, recall, and F1-score.**

| Model | Prediction score used for classification | Threshold selection method | Dataset used to determine the threshold | Training-set threshold | Application to evaluation datasets |
| --- | --- | --- | --- | --- | --- |
| Random forest | Predicted probability of Event_Yes | Youden index from training-set ROC analysis | Training set | 0.075 | The same threshold was applied unchanged to the internal validation set and external cohort |
| Gradient boosting decision tree | Predicted probability of Event_Yes | Youden index from training-set ROC analysis | Training set | 0.092 | The same threshold was applied unchanged to the internal validation set and external cohort |
| XGBoost | Predicted probability of Event_Yes | Youden index from training-set ROC analysis | Training set | 0.045 | The same threshold was applied unchanged to the internal validation set and external cohort |
| Naive Bayes | Predicted probability of Event_Yes | Youden index from training-set ROC analysis | Training set | 0.056 | The same threshold was applied unchanged to the internal validation set and external cohort |
| AdaBoost | Predicted probability of Event_Yes | Youden index from training-set ROC analysis | Training set | 0.403 | The same threshold was applied unchanged to the internal validation set and external cohort |
| Support vector machine | Predicted probability of Event_Yes | Youden index from training-set ROC analysis | Training set | 0.111 | The same threshold was applied unchanged to the internal validation set and external cohort |
| Cox regression | Predicted 3-year event risk | Youden index from training-set ROC analysis | Training set | 0.086 | The same threshold was applied unchanged to the internal validation set and external cohort |

The classification threshold for each model was determined in the training set by maximizing the Youden index, defined as sensitivity+specificity-1. Predicted probabilities or risk scores greater than or equal to the training-set threshold were classified as Event-Yes, whereas values below the threshold were classified as Event-No. The same model-specific threshold was then applied unchanged to the internal validation set and external cohort to calculate accuracy, precision, recall, F1-score, sensitivity, and specificity. XGBoost, eXtreme gradient boosting; AdaBoost, adaptive boosting; ROC, receiver operating characteristic curve.

**Supplementary Table 3. Definitions of performance metrics used for model comparison.**

| Metric | Prediction used | Time point or follow-up structure | Threshold required | Definition and interpretation |
| --- | --- | --- | --- | --- |
| Time-dependent AUC | Continuous predicted probability, risk score, or Cox linear predictor | Primary evaluation at 3 years | No | Measures time-specific discrimination, indicating the ability of a model to distinguish participants who developed FPG-defined incident prediabetes by a given time point from those who remained event-free at that time while accounting for censored follow-up data. |
| C-index | Continuous predicted probability, risk score, or Cox linear predictor | Complete follow-up time with censoring information | No | Measures concordance between predicted risk and observed time-to-event outcome. Higher values indicate better ranking of participants according to event risk. |
| Accuracy | Threshold-based predicted class | Binary classification of observed FPG-defined incident prediabetes status | Yes | Proportion of correctly classified participants among all evaluable participants: (true positives + true negatives) / total participants. |
| Precision | Threshold-based predicted class | Binary classification of observed FPG-defined incident prediabetes status | Yes | Positive predictive value: proportion of true events among participants predicted as Event_Yes. |
| Recall | Threshold-based predicted class | Binary classification of observed FPG-defined incident prediabetes status | Yes | Sensitivity: proportion of actual Event_Yes participants correctly identified by the model. |
| F1-score | Threshold-based predicted class | Binary classification of observed FPG-defined incident prediabetes status | Yes | Harmonic mean of precision and recall, calculated as 2 × precision × recall / (precision + recall). |

Predicted probabilities or risk scores greater than or equal to the training-set threshold were classified as Event-Yes, whereas values below the threshold were classified as Event-No. AUC, area under the curve; FPG, fasting plasma glucose.

**Supplementary Table 4. Baseline comparability between the training and internal validation sets.**

| Variables | Training set  (n = 6888) | Internal validation set (n = 3704) | P value |
| --- | --- | --- | --- |
| Male sex, n (%) | 4513 (65.5) | 2440 (65.92) | 0.73 |
| Smoking history, n (%) | 1586 (23.0) | 894 (24.11) | 0.21 |
| Drinking history, n (%) | 1524 (22.11) | 843 (22.82) | 0.47 |
| Family history of diabetes, n (%) | 401 (5.82) | 229 (6.18) | 0.48 |
| Age, years | 40.9±11.12 | 40.8±11.11 | 0.55 |
| BMI, kg/m^2^ | 23.3±3.23 | 23.4±3.26 | 0.39 |
| SBP, mmHg | 118±14.92 | 118±14.94 | 0.37 |
| DBP, mmHg | 74.1±10.23 | 74.1±10.31 | 0.78 |
| FPG, mmol/L | 4.83±0.48 | 4.82±0.48 | 0.35 |
| Cholesterol, mmol/L | 4.71±0.88 | 4.72±0.88 | 0.43 |
| Triglyceride, mmol/L | 1.38±1.05 | 1.38±1.04 | 0.81 |
| HDL, mmol/L | 1.35±0.30 | 1.35±0.29 | 0.89 |
| LDL, mmol/L | 2.70±0.68 | 2.71±0.68 | 0.56 |
| ALT, U/L | 25.2±21.44 | 25.3±22.31 | 0.75 |
| AST, U/L | 24.6±10.52 | 24.6±10.41 | 0.93 |
| BUN, mmol/L | 4.70±1.16 | 4.67±1.17 | 0.19 |
| Creatinine, μmol/L | 73.4±15.32 | 73.3±15.11 | 0.88 |

Data are shown as means ± standard deviation for normally distributed variables and as percentages for categorical variables. BMI, body mass index; SBP, systolic blood pressure; DBP, diastolic blood pressure; FPG, fasting plasma glucose; HDL, high-density lipoprotein; LDL, low-density lipoprotein; ALT, alanine aminotransferase; AST, aspartate aminotransferase; BUN, blood urea nitrogen.

**Supplementary Table 5. Baseline characteristics of participants with and without FPG-defined incident prediabetes in the training set.**

| Variables | No FPG-defined incident prediabetes  (n = 6035) | FPG-defined incident prediabetes (n = 853) | P value |
| --- | --- | --- | --- |
| Male sex, n (%) | 3869 (64.1) | 644 (75.5) | < 0.01 |
| Smoking history, n (%) | 1323 (21.9) | 263 (30.8) | < 0.01 |
| Drinking history, n (%) | 1300 (21.5) | 224 (26.3) | < 0.01 |
| Family history of diabetes, n (%) | 326 (5.4) | 75 (8.8) | < 0.01 |
| Age, years | 40.27±10.83 | 45.4±11.84 | < 0.01 |
| BMI, kg/m^2^ | 23.15±3.18 | 24.63±3.33 | < 0.01 |
| SBP, mmHg | 117.1±14.72 | 123.4±15.31 | < 0.01 |
| DBP, mmHg | 73.56±10.1 | 78.08±10.32 | < 0.01 |
| FPG, mmol/L | 4.8±0.48 | 5.05±0.4 | < 0.01 |
| Cholesterol, mmol/L | 4.69±0.88 | 4.87±0.88 | < 0.01 |
| Triglyceride, mmol/L | 1.33±1.03 | 1.71±1.1 | < 0.01 |
| HDL, mmol/L | 1.36±0.3 | 1.29±0.3 | < 0.01 |
| LDL, mmol/L | 2.68±0.68 | 2.81±0.68 | < 0.01 |
| ALT, U/L | 24.44±20.32 | 30.24±27.55 | < 0.01 |
| AST, U/L | 24.42±10.08 | 26.25±12.86 | < 0.01 |
| BUN, mmol/L | 4.67±1.16 | 4.9±1.19 | < 0.01 |
| Creatinine, μmol/L | 73.16±15.43 | 75.02±14.45 | < 0.01 |

Data are shown as means ± standard deviation for normally distributed variables and percentages for categorical variables. BMI, body mass index; SBP, systolic blood pressure; DBP, diastolic blood pressure; FPG, fasting plasma glucose; HDL, high-density lipoprotein; LDL, low-density lipoprotein; ALT, alanine aminotransferase; AST, aspartate aminotransferase; BUN, blood urea nitrogen.

**Supplementary Table 6. Baseline comparability between the development cohort and the external cohort.**

| Variable | Development set  (n = 10592) | External cohort  (n = 671) | P value |
| --- | --- | --- | --- |
| Male sex, n (%) | 6953 (65.6) | 455 (67.8) | 0.269 |
| Smoking history, n (%) | 2480 (23.4) | 165 (24.6) | 0.516 |
| Drinking history, n (%) | 2367 (22.3) | 143 (21.3) | 0.564 |
| Family history of diabetes, n (%) | 630 (5.9) | 35 (5.2) | 0.487 |
| Age, years | 40.86±11.11 | 41.72±11.43 | 0.051 |
| BMI, kg/m^2^ | 23.35±3.24 | 23.20±3.12 | 0.237 |
| SBP, mmHg | 117.97±14.92 | 117.46±14.30 | 0.384 |
| DBP, mmHg | 74.10±10.26 | 73.49±10.01 | 0.131 |
| FPG, mmol/L | 4.83±0.48 | 4.82±0.48 | 0.621 |
| Cholesterol, mmol/L | 4.71±0.88 | 4.66±0.82 | 0.104 |
| Triglyceride, mmol/L | 1.38±1.04 | 1.39±1.07 | 0.833 |
| HDL, mmol/L | 1.35±0.30 | 1.34±0.30 | 0.473 |
| LDL, mmol/L | 2.70±0.68 | 2.67±0.64 | 0.268 |
| ALT, U/L | 25.21±21.74 | 24.04±16.69 | 0.172 |
| AST, U/L | 24.64±10.44 | 24.33±7.74 | 0.456 |
| BUN, mmol/L | 4.69±1.16 | 4.66±1.14 | 0.496 |
| Creatinine, μmol/L | 73.38±15.24 | 74.23±15.01 | 0.161 |

Data are shown as means ± standard deviation for normally distributed variables and percentages for categorical variables. BMI, body mass index; SBP, systolic blood pressure; DBP, diastolic blood pressure; FPG, fasting plasma glucose; HDL, high-density lipoprotein; LDL, low-density lipoprotein; ALT, alanine aminotransferase; AST, aspartate aminotransferase; BUN, blood urea nitrogen.

**Supplementary Table 7. Baseline characteristics of participants with and without FPG-defined incident prediabetes in the external cohort.**

| Variable | No FPG-defined incident prediabetes  (n = 596) | FPG-defined incident prediabetes (n = 75) | P value |
| --- | --- | --- | --- |
| Male sex, n (%) | 401 (67.3) | 54 (72.0) | 0.488 |
| Smoking history, n (%) | 142 (23.8) | 23 (30.7) | 0.248 |
| Drinking history, n (%) | 124 (20.8) | 19 (25.3) | 0.452 |
| Family history of diabetes, n (%) | 26 (4.4) | 9 (12.0) | 0.011 |
| Age, years | 41.30±11.10 | 45.13±13.35 | 0.006 |
| BMI, kg/m^2^ | 23.06±3.06 | 24.26±3.42 | 0.002 |
| SBP, mmHg | 116.97±14.25 | 121.36±14.15 | 0.012 |
| DBP, mmHg | 73.08±9.88 | 76.71±10.53 | 0.003 |
| FPG, mmol/L | 4.80±0.49 | 4.96±0.43 | 0.007 |
| Cholesterol, mmol/L | 4.64±0.82 | 4.83±0.76 | 0.050 |
| Triglyceride, mmol/L | 1.33±1.02 | 1.80±1.35 | <0.001 |
| HDL, mmol/L | 1.34±0.30 | 1.32±0.29 | 0.509 |
| LDL, mmol/L | 2.66±0.64 | 2.78±0.62 | 0.123 |
| ALT, U/L | 23.42±15.74 | 28.97±22.36 | 0.007 |
| AST, U/L | 24.09±7.74 | 26.26±7.49 | 0.022 |
| BUN, mmol/L | 4.65±1.14 | 4.74±1.18 | 0.536 |
| Creatinine, μmol/L | 74.24±14.92 | 74.11±15.86 | 0.944 |

Data are shown as means ± standard deviation for normally distributed variables and percentages for categorical variables. BMI, body mass index; SBP, systolic blood pressure; DBP, diastolic blood pressure; FPG, fasting plasma glucose; HDL, high-density lipoprotein; LDL, low-density lipoprotein; ALT, alanine aminotransferase; AST, aspartate aminotransferase; BUN, blood urea nitrogen.

**Supplementary Table 8. Coefficient of variable selection from LASSO regression.**

| Variables | Coefficient |
| --- | --- |
| Age, years | 0.017981416 |
| BMI | 0.049698212 |
| DBP | 0.014419721 |
| FPG | 1.661422879 |
| Triglyceride | 0.026002426 |
| ALT | 0.001978101 |
| BUN | 0.009866322 |
| Sex | 0.056086177 |
| Smoking history | 0.062347208 |
| Drinking history | -0.041158677 |
| Family history of diabetes | 0.154996035 |

BMI, body mass index; DBP, diastolic blood pressure; FPG, fasting plasma glucose; ALT, alanine aminotransferase; BUN, blood urea nitrogen.

**Supplementary Table 9. Performance of six machine-learning models and the initial proportional-hazards Cox model in the training set, internal validation set, and external cohort.**

| Cohort | Model | t-AUC | Accuracy | Precision | Recall | F1-score | C-index (95% CI) |
| --- | --- | --- | --- | --- | --- | --- | --- |
| Training set | RF | 0.936 | 0.979 | 0.984 | 0.993 | 0.975 | 0.936(0.931 - 0.942) |
|  | GBDT | 0.793 | 0.700 | 0.250 | 0.715 | 0.371 | 0.772(0.755 - 0.789) |
|  | XGBoost | 0.789 | 0.704 | 0.251 | 0.699 | 0.369 | 0.767(0.751 - 0.784) |
|  | NB | 0.707 | 0.612 | 0.201 | 0.719 | 0.314 | 0.700(0.681 - 0.719) |
|  | AdaBoost | 0.814 | 0.715 | 0.273 | 0.782 | 0.405 | 0.804(0.789 - 0.817) |
|  | SVM | 0.814 | 0.900 | 0.582 | 0.679 | 0.627 | 0.794(0.777 - 0.812) |
|  | Cox | 0.799 | 0.717 | 0.248 | 0.783 | 0.377 | 0.766(0.751 - 0.783) |
| Internal validation set | RF | 0.717 | 0.710 | 0.220 | 0.583 | 0.320 | 0.704(0.675 - 0.730) |
|  | GBDT | 0.753 | 0.672 | 0.207 | 0.638 | 0.313 | 0.731(0.704 - 0.757) |
|  | XGBoost | 0.755 | 0.669 | 0.207 | 0.645 | 0.314 | 0.732(0.705 - 0.760) |
|  | NB | 0.705 | 0.579 | 0.176 | 0.703 | 0.281 | 0.678(0.653 - 0.704) |
|  | AdaBoost | 0.756 | 0.683 | 0.211 | 0.624 | 0.316 | 0.727(0.701 - 0.752) |
|  | SVM | 0.553 | 0.772 | 0.203 | 0.325 | 0.250 | 0.563(0.526 - 0.598) |
|  | Cox | 0.780 | 0.700 | 0.228 | 0.777 | 0.352 | 0.756(0.732 - 0.781) |
| External cohort | RF | 0.685 | 0.484 | 0.153 | 0.801 | 0.258 | 0.675(0.600 - 0.753) |
|  | GBDT | 0.711 | 0.717 | 0.208 | 0.547 | 0.301 | 0.681(0.611 - 0.746) |
|  | XGBoost | 0.678 | 0.501 | 0.161 | 0.827 | 0.270 | 0.672(0.609 - 0.734) |
|  | NB | 0.675 | 0.581 | 0.166 | 0.680 | 0.266 | 0.663(0.597 - 0.728) |
|  | AdaBoost | 0.465 | 0.541 | 0.158 | 0.720 | 0.260 | 0.549(0.478 - 0.624) |
|  | SVM | 0.593 | 0.683 | 0.177 | 0.502 | 0.261 | 0.594(0.548 - 0.639) |
|  | Cox | 0.769 | 0.588 | 0.180 | 0.882 | 0.299 | 0.734(0.660 - 0.796) |

FPG, fasting plasma glucose; RF, random forest; GBDT, gradient boosting decision tree; XGBoost, eXtreme gradient boosting; NB, naive Bayes; AdaBoost, adaptive boosting; SVM, support vector machine; Cox, Cox proportional hazards regression model; t-AUC, time-dependent area under the curve; CI, confidence interval.

**Supplementary Table 10.** **Multivariable analysis using the initial proportional-hazards Cox model in the training set.**

| Variables | B | HR (95% CI) | P value |
| --- | --- | --- | --- |
| Age, years | 0.0191 | 1.02 (1.01 - 1.03) | 0.001 |
| BMI, kg/m^2^ | 0.0514 | 1.05 (1.03 - 1.08) | 0.001 |
| DBP, mmHg | 0.0155 | 1.02 (1.01 - 1.02) | 0.001 |
| FPG, mmol/L | 1.7314 | 5.65 (4.71 - 6.78) | 0.001 |
| Triglyceride | 0.0289 | 1.03 (0.97 - 1.09) | 0.326 |
| ALT | 0.0025 | 1.00 (1.00 - 1.01) | 0.089 |
| BUN | 0.0233 | 1.02 (0.97 - 1.08) | 0.434 |
| Sex, yes vs no | 0.1018 | 1.11 (0.92 - 1.33) | 0.281 |
| Smoking history, yes vs no | 0.0949 | 1.10 (0.94 - 1.29) | 0.248 |
| Drinking history, yes vs no | -0.1299 | 0.88 (0.75 - 1.03) | 0.117 |
| Family history of diabetes, yes vs no | 0.2422 | 1.27 (1.00 - 1.62) | 0.047 |

BMI, body mass index; DBP, diastolic blood pressure; FPG, fasting plasma glucose; ALT, alanine aminotransferase; BUN, blood urea nitrogen. B, regression coefficient β; HR, hazard ratio; CI, confidence interval.

**Supplementary Table 11.** **Assessment of the proportional hazards assumption for the initial Cox model in the training set.**

| Variable | Chi-square | DF | P value |
| --- | --- | --- | --- |
| Age, years | 3.609 | 1 | 0.057 |
| BMI, kg/m^2^ | 0.051 | 1 | 0.821 |
| DBP, mmHg | 0.031 | 1 | 0.861 |
| FPG, mmol/L | 15.666 | 1 | <0.001 |
| Family history of diabetes | 1.857 | 1 | 0.173 |
| GLOBAL | 24.029 | 5 | <0.001 |

The GLOBAL test evaluates the proportional hazards assumption for the overall model. BMI, body mass index; DBP, diastolic blood pressure; FPG, fasting plasma glucose; DF, degrees of freedom.

**Supplementary Table 12. Discrimination and calibration of alternative Cox models in the internal validation set and external cohort.**

| Cohort | Horizon, y | At risk / events | Model | AUC (95% CI) | C-index (95% CI) | Brier (95% CI) | Observed risk (95% CI) | Predicted risk | O/E |
| --- | --- | --- | --- | --- | --- | --- | --- | --- | --- |
| Internal validation set | 3 | 1,724 / 202 | M0 | 0.777 (0.744-0.811) | 0.753 (0.728-0.777) | 0.064 (0.057-0.072) | 0.074 (0.064-0.085) | 0.083 | 0.894 |
| Internal validation set | 3 | 1,724 / 202 | M1 | 0.779 (0.746-0.813) | 0.754 (0.730-0.778) | 0.064 (0.057-0.072) | 0.074 (0.064-0.085) | 0.083 | 0.898 |
| Internal validation set | 3 | 1,724 / 202 | M2 | 0.778 (0.744-0.812) | 0.754 (0.729-0.778) | 0.064 (0.057-0.072) | 0.074 (0.064-0.085) | 0.083 | 0.898 |
| Internal validation set | 3 | 1,724 / 202 | M3 | 0.779 (0.745-0.812) | 0.754 (0.729-0.778) | 0.064 (0.057-0.072) | 0.074 (0.064-0.085) | 0.083 | 0.899 |
| Internal validation set | 4 | 680 / 324 | M0 | 0.758 (0.724-0.792) | 0.753 (0.728-0.777) | 0.125 (0.113-0.136) | 0.171 (0.151-0.190) | 0.214 | 0.797 |
| Internal validation set | 4 | 680 / 324 | M1 | 0.758 (0.724-0.793) | 0.754 (0.730-0.778) | 0.125 (0.113-0.136) | 0.171 (0.151-0.190) | 0.213 | 0.803 |
| Internal validation set | 4 | 680 / 324 | M2 | 0.758 (0.724-0.792) | 0.753 (0.729-0.777) | 0.124 (0.113-0.136) | 0.171 (0.151-0.190) | 0.212 | 0.805 |
| Internal validation set | 4 | 680 / 324 | M3 | 0.759 (0.724-0.793) | 0.754 (0.729-0.778) | 0.125 (0.113-0.136) | 0.171 (0.151-0.190) | 0.211 | 0.808 |
| Internal validation set | 5 | 75 / 419 | M0 | 0.740 (0.669-0.811) | 0.753 (0.728-0.777) | 0.211 (0.177-0.246) | 0.425 (0.371-0.473) | 0.452 | 0.940 |
| Internal validation set | 5 | 75 / 419 | M1 | 0.742 (0.671-0.812) | 0.754 (0.730-0.778) | 0.211 (0.177-0.246) | 0.425 (0.371-0.473) | 0.447 | 0.949 |
| Internal validation set | 5 | 75 / 419 | M2 | 0.739 (0.669-0.810) | 0.750 (0.726-0.775) | 0.208 (0.177-0.239) | 0.425 (0.371-0.473) | 0.449 | 0.945 |
| Internal validation set | 5 | 75 / 419 | M3 | 0.740 (0.669-0.810) | 0.752 (0.727-0.777) | 0.208 (0.177-0.240) | 0.425 (0.371-0.473) | 0.447 | 0.950 |
| External cohort | 3 | 309 / 34 | M0 | 0.737 (0.653-0.821) | 0.705 (0.639-0.771) | 0.065 (0.046-0.085) | 0.075 (0.049-0.099) | 0.081 | 0.915 |
| External cohort | 3 | 309 / 34 | M1 | 0.743 (0.660-0.826) | 0.709 (0.643-0.775) | 0.065 (0.045-0.084) | 0.075 (0.049-0.099) | 0.081 | 0.920 |
| External cohort | 3 | 309 / 34 | M2 | 0.739 (0.653-0.824) | 0.706 (0.640-0.773) | 0.065 (0.046-0.084) | 0.075 (0.049-0.099) | 0.081 | 0.920 |
| External cohort | 3 | 309 / 34 | M3 | 0.740 (0.655-0.825) | 0.707 (0.640-0.774) | 0.065 (0.045-0.084) | 0.075 (0.049-0.099) | 0.081 | 0.922 |
| External cohort | 4 | 122 / 59 | M0 | 0.754 (0.672-0.835) | 0.705 (0.639-0.771) | 0.133 (0.104-0.162) | 0.191 (0.140-0.240) | 0.211 | 0.907 |
| External cohort | 4 | 122 / 59 | M1 | 0.755 (0.674-0.836) | 0.709 (0.643-0.775) | 0.133 (0.103-0.162) | 0.191 (0.140-0.240) | 0.209 | 0.915 |
| External cohort | 4 | 122 / 59 | M2 | 0.754 (0.673-0.836) | 0.705 (0.639-0.772) | 0.133 (0.104-0.162) | 0.191 (0.140-0.240) | 0.209 | 0.917 |
| External cohort | 4 | 122 / 59 | M3 | 0.755 (0.674-0.836) | 0.708 (0.642-0.774) | 0.133 (0.104-0.162) | 0.191 (0.140-0.240) | 0.208 | 0.921 |
| External cohort | 5 | 20 / 73 | M0 | 0.655 (0.474-0.837) | 0.705 (0.639-0.771) | 0.241 (0.163-0.320) | 0.425 (0.280-0.541) | 0.448 | 0.949 |
| External cohort | 5 | 20 / 73 | M1 | 0.669 (0.491-0.848) | 0.709 (0.643-0.775) | 0.239 (0.163-0.316) | 0.425 (0.280-0.541) | 0.443 | 0.959 |
| External cohort | 5 | 20 / 73 | M2 | 0.677 (0.498-0.855) | 0.702 (0.636-0.769) | 0.231 (0.159-0.304) | 0.425 (0.280-0.541) | 0.446 | 0.954 |
| External cohort | 5 | 20 / 73 | M3 | 0.677 (0.500-0.855) | 0.707 (0.641-0.772) | 0.232 (0.159-0.304) | 0.425 (0.280-0.541) | 0.443 | 0.959 |

AUC represents cumulative/dynamic time-dependent discrimination, and Brier scores were estimated using inverse-probability-of-censoring weighting. The C-index represents Harrell concordance over the complete observed follow-up using the predicted risk at each horizon as the prognostic marker. Observed risk was estimated by the Kaplan–Meier method; O/E was calculated as observed risk divided by mean predicted risk. All M0–M3 models were evaluated using identical procedures within this analysis; these estimates are intended for within-table model comparison and should not be directly compared with horizon-specific estimates in subsequent sensitivity analyses. AUC, area under the curve; O/E, observed-to-expected ratio; CI, confidence interval.

**Supplementary Table 13. Regression coefficients and parameterization of the final extended Cox model (M2).**

| Term | Centering / coding | Beta | SE | HR | HR 95% CI | P value | Interpretation |
| --- | --- | --- | --- | --- | --- | --- | --- |
| Age | Age - 40.9063589 | 0.019339 | 0.002948 | 1.0195 | 1.0137-1.0254 | 5.40E-11 | Per 1-year higher age |
| BMI | BMI - 23.3298824 | 0.062122 | 0.010922 | 1.0641 | 1.0416-1.0871 | 1.29E-8 | Per 1-kg/m2 higher BMI |
| DBP | DBP - 74.1231127 | 0.016951 | 0.003466 | 1.0171 | 1.0102-1.0240 | 1.00E-6 | Per 1-mmHg higher DBP |
| FPG main effect | FPG - 4.8301524 | 1.845396 | 0.097776 | 6.3306 | 5.2266-7.6679 | 1.88E-79 | Per 1-mmol/L higher FPG at 3 years |
| Family history | 1=present; 0=absent | 0.220771 | 0.121332 | 1.2470 | 0.9831-1.5818 | 0.0688 | Present versus absent |
| FPG x log(t/3) | (FPG - 4.8301524) x log(t/3) | -1.493193 | 0.332847 | NA | NA | 7.25E-6 | Change in FPG log-HR over log time |

Continuous predictors were centered at their training-cohort means. The time-dependent linear predictor was η(u) = 0.0193388Age_c + 0.0621223BMI_c + 0.0169510DBP_c + 0.2207706Family_history + [1.8453957 − 1.4931933 log(u/3)]FPG_c, where u denotes follow-up time in years. The FPG main effect is defined at the 3-year reference time; the interaction coefficient should not be interpreted as a stand-alone clinical HR. BMI, body mass index; DBP, diastolic blood pressure; FPG, fasting plasma glucose; β, regression coefficient; SE, standard error; HR, hazard ratio; CI, confidence interval; M2, the final extended Cox model incorporating FPG × log(t/3).

**Supplementary Table 14. Baseline cumulative hazard and survival estimates for absolute-risk prediction with the final extended Cox model (M2).**

| Horizon, years | Role | Last event time <= horizon | H0(t) | S0(t) | Reference-profile risk |
| --- | --- | --- | --- | --- | --- |
| 3 | Primary | 2.997946612 | 0.052924071 | 0.948452024 | 0.051547976 |
| 4 | Primary | 4.000000000 | 0.172170002 | 0.841836047 | 0.158163953 |
| 5 | Exploratory | 4.996577687 | 0.524108707 | 0.592082848 | 0.407917152 |

H₀(t) denotes the baseline cumulative hazard and S₀(t) = exp[−H₀(t)]. For M2, H(t|X) = Σuⱼ≤t ΔH₀(uⱼ)exp[η(uⱼ)] and Risk(t|X) = 1 − exp[−H(t|X)], where η(u) is defined in Supplementary Table 13. Exact prediction requires the locked Efron baseline-hazard increments at all 429 distinct training event times; the conventional fixed-linear-predictor Cox formula is not applicable to M2. H₀(t), baseline cumulative hazard; S₀(t), baseline survival; M2, the final extended Cox model.

**Supplementary Table 15. Discrimination and calibration of the FPG-only M2, non-FPG clinical, and full five-predictor M2 models in the internal validation set and external cohort.**

| Cohort | Horizon, y | Model | AUC | C-index | Brier | Observed risk | Predicted risk | O/E |
| --- | --- | --- | --- | --- | --- | --- | --- | --- |
| Internal validation set | 3 | FPG-only M2 | 0.759 | 0.745 | 0.0641 | 0.074 | 0.085 | 0.874 |
| Internal validation set | 3 | Non-FPG clinical Cox | 0.662 | 0.656 | 0.0677 | 0.074 | 0.078 | 0.950 |
| Internal validation set | 3 | Full five-predictor M2 | 0.778 | 0.768 | 0.0642 | 0.074 | 0.083 | 0.898 |
| Internal validation set | 4 | FPG-only M2 | 0.741 | 0.736 | 0.1265 | 0.171 | 0.225 | 0.758 |
| Internal validation set | 4 | Non-FPG clinical Cox | 0.644 | 0.649 | 0.1367 | 0.171 | 0.189 | 0.902 |
| Internal validation set | 4 | Full five-predictor M2 | 0.755 | 0.755 | 0.1244 | 0.171 | 0.212 | 0.805 |
| Internal validation set | 5 | FPG-only M2 | 0.731 | 0.734 | 0.2065 | 0.425 | 0.488 | 0.869 |
| Internal validation set | 5 | Non-FPG clinical Cox | 0.675 | 0.648 | 0.2233 | 0.425 | 0.407 | 1.042 |
| Internal validation set | 5 | Full five-predictor M2 | 0.739 | 0.751 | 0.2070 | 0.425 | 0.449 | 0.945 |
| External cohort | 3 | FPG-only M2 | 0.748 | 0.720 | 0.0650 | 0.075 | 0.084 | 0.882 |
| External cohort | 3 | Non-FPG clinical Cox | 0.619 | 0.604 | 0.0681 | 0.075 | 0.077 | 0.973 |
| External cohort | 3 | Full five-predictor M2 | 0.750 | 0.722 | 0.0649 | 0.075 | 0.081 | 0.920 |
| External cohort | 4 | FPG-only M2 | 0.765 | 0.704 | 0.1349 | 0.191 | 0.224 | 0.854 |
| External cohort | 4 | Non-FPG clinical Cox | 0.643 | 0.605 | 0.1510 | 0.191 | 0.186 | 1.028 |
| External cohort | 4 | Full five-predictor M2 | 0.764 | 0.705 | 0.1329 | 0.191 | 0.209 | 0.917 |
| External cohort | 5 | FPG-only M2 | 0.636 | 0.704 | 0.2323 | 0.425 | 0.486 | 0.874 |
| External cohort | 5 | Non-FPG clinical Cox | 0.669 | 0.608 | 0.2237 | 0.425 | 0.404 | 1.052 |
| External cohort | 5 | Full five-predictor M2 | 0.667 | 0.702 | 0.2265 | 0.425 | 0.446 | 0.954 |

All models were fitted in the original training cohort (n = 6,888; 853 events) and applied without refitting or recalibration. The FPG-only M2 included baseline FPG and FPG × log(t/3); the non-FPG clinical Cox model included age, BMI, DBP, and family history of diabetes; the full five-predictor M2 included all five predictors and FPG × log(t/3). AUC and Brier scores were estimated using IPCW, and the C-index was calculated after administrative truncation at each horizon. Observed risk was estimated using the Kaplan–Meier method, and O/E was calculated as observed risk divided by mean predicted risk. AUC, area under the curve; C-index, concordance index; IPCW, inverse probability of censoring weighting; O/E, observed-to-expected ratio; FPG, fasting plasma glucose; M2, the final extended Cox model.

**Supplementary Table 16. Incremental predictive value of the full five-predictor M2 model beyond the FPG-only M2 model in the internal validation set and external cohort.**

| Cohort | Horizon, y | Delta AUC | Delta C-index | Delta Brier | Delta absolute calibration error |
| --- | --- | --- | --- | --- | --- |
| Internal validation | 3 | +0.018 | +0.022 | +0.00015 | -0.00228 |
| Internal validation | 4 | +0.013 | +0.019 | -0.00210 | -0.01322 |
| Internal validation | 5 | +0.008 | +0.016 | +0.00046 | -0.03911 |
| External cohort | 3 | +0.002 | +0.002 | -0.00004 | -0.00348 |
| External cohort | 4 | -0.001 | +0.001 | -0.00200 | -0.01526 |
| External cohort | 5 | +0.030 | -0.001 | -0.00577 | -0.04071 |

Differences were calculated as the full five-predictor M2 model minus the FPG-only M2 model using unrounded estimates. Positive ΔAUC and ΔC-index values and negative ΔBrier and Δabsolute calibration-error values favor the full model. Minor discrepancies from subtraction of displayed values may result from rounding. FPG, fasting plasma glucose; M2, extended Cox model with FPG × log(t/3); AUC, area under the curve; Δ, between-model difference.

**Supplementary Table 17. Construction of the sensitivity-analysis cohort after removal of the post-baseline upper-FPG exclusion criterion.**

| Cohort / stage | n | Events | Definition |
| --- | --- | --- | --- |
| Primary development cohort | 10,592 | 1,287 | Original complete-case cohort |
| Re-included participants | 50 | 50 | 50 with final FPG >=7.0 mmol/L |
| Expanded development cohort | 10,642 | 1,337 | No post-baseline upper-FPG exclusion |
| Expanded training set | 6,920 | 885 | M2 refitted; predictor set and FPG time interaction frozen |
| Expanded internal validation set | 3,722 | 452 | Locked sensitivity coefficients; no recalibration |

The five-predictor M2 structure and FPG × log(t/3) interaction were retained without renewed variable selection. Coefficients were re-estimated in the expanded training set and applied without recalibration to the expanded internal validation set. FPG, fasting plasma glucose; M2, the final extended Cox model.

**Supplementary Table 18. Stability of model coefficients and time-varying FPG effects after removal of the post-baseline upper-FPG exclusion criterion.**

| Model quantity | Primary M2 | Sensitivity M2 | Relative change |
| --- | --- | --- | --- |
| Age, per year | 1.020 (1.014-1.025) | 1.020 (1.015-1.026) | +3.9% beta |
| BMI, per kg/m2 | 1.064 (1.042-1.087) | 1.067 (1.045-1.090) | +4.6% beta |
| DBP, per mmHg | 1.017 (1.010-1.024) | 1.017 (1.011-1.024) | +1.4% beta |
| FPG at 3 years, per mmol/L | 6.331 (5.227-7.668) | 6.139 (5.087-7.408) | -1.7% beta |
| Family history, yes vs no | 1.247 (0.983-1.582) | 1.233 (0.974-1.561) | -5.1% beta |
| FPG x log(t/3), beta (SE) | -1.493 (0.333) | -1.421 (0.327) | +4.8% beta |
| FPG HR at 2 years | 11.598 (8.007-16.799) | 10.923 (7.590-15.718) | -5.82% HR |
| FPG HR at 4 years | 4.120 (3.292-5.155) | 4.079 (3.276-5.079) | -1.00% HR |
| FPG HR at 5 years* | 2.952 (2.121-4.109) | 2.970 (2.149-4.105) | +0.61% HR |

Relative changes in regression coefficients were calculated as (β_sensitivity − β_primary)/|β_primary| × 100%, whereas changes in time-specific FPG HRs were calculated on the HR scale. BMI, body mass index; DBP, diastolic blood pressure; FPG, fasting plasma glucose; β, regression coefficient; SE, standard error; HR, hazard ratio; CI, confidence interval.

**Supplementary Table 19. Internal-validation performance of the primary and sensitivity M2 models after removal of the post-baseline upper-FPG exclusion criterion.**

| Horizon, y | Analysis | AUC | C-index | Brier | Observed risk | Predicted risk | O/E |
| --- | --- | --- | --- | --- | --- | --- | --- |
| 3 | Primary | 0.778 | 0.768 | 0.064 | 0.074 | 0.083 | 0.898 |
| 3 | Sensitivity | 0.780 | 0.770 | 0.066 | 0.077 | 0.085 | 0.903 |
| 4 | Primary | 0.755 | 0.755 | 0.124 | 0.171 | 0.212 | 0.805 |
| 4 | Sensitivity | 0.760 | 0.759 | 0.126 | 0.175 | 0.216 | 0.813 |
| 5 | Primary | 0.739 | 0.751 | 0.207 | 0.425 | 0.449 | 0.945 |
| 5 | Sensitivity | 0.747 | 0.753 | 0.205 | 0.443 | 0.458 | 0.968 |

The sensitivity endpoint was any final follow-up FPG ≥5.6 mmol/L. AUC and Brier scores were estimated using IPCW, and the C-index was calculated after administrative truncation at each prediction horizon. Observed risk was estimated using the Kaplan-Meier method, and O/E was calculated as observed risk divided by mean predicted risk. AUC, area under the curve; C-index, concordance index; IPCW, inverse probability of censoring weighting; O/E, observed-to-expected ratio; FPG, fasting plasma glucose; M2, the final extended Cox model.

**Supplementary Table 20. Construction of the expanded five-predictor cohort for re-estimation of the M2 model.**

| Cohort / stage | n | Events | Definition |
| --- | --- | --- | --- |
| Source cohort | 211,833 | - | Source records |
| Baseline FPG <5.6 mmol/L | 185,586 | - | Baseline normal fasting glucose |
| Corrected strict complete-case cohort | 10,642 | 1,337 | All 17 candidate variables complete; no upper follow-up-FPG exclusion |
| Expanded five-predictor cohort | 185,563 | 21,913 | Complete age, BMI, DBP, FPG, family history, outcome, and follow-up |
| Expanded training set | 120,619 | 14,259 | M2 structure frozen and coefficients re-estimated |
| Expanded internal-validation set | 64,944 | 7,654 | Locked expanded-cohort coefficients; no recalibration |
| External cohort | 671 | 75 | Supplied external cohort |

The expanded cohort required complete data only for age, BMI, DBP, FPG, family history of diabetes, outcome status, and follow-up time, without an upper post-baseline FPG exclusion. The M2 structure was retained without renewed variable selection and re-estimated in the expanded training cohort. BMI, body mass index; DBP, diastolic blood pressure; FPG, fasting plasma glucose; M2, extended Cox model with FPG × log(t/3).

**Supplementary Table 21. Regression coefficients and time-specific FPG effects after re-estimation of M2 in the expanded five-predictor cohort.**

| Model quantity | Corrected complete-case M2 | Expanded five-predictor M2 |
| --- | --- | --- |
| Age, per year | 1.020 (1.015-1.026) | 1.021 (1.019-1.022) |
| BMI, per kg/m2 | 1.067 (1.045-1.090) | 1.072 (1.067-1.078) |
| DBP, per mmHg | 1.017 (1.011-1.024) | 1.012 (1.011-1.014) |
| FPG at 3 years, per mmol/L | 6.139 (5.087-7.408) | 4.819 (4.610-5.038) |
| Family history, yes vs no | 1.233 (0.974-1.561) | 1.149 (1.034-1.277) |
| FPG x log(t/3), beta (SE) | -1.421 (0.327) | -1.070 (0.074) |
| FPG HR at 2 years | 10.923 (7.590-15.718) | 7.436 (6.837-8.088) |
| FPG HR at 4 years | 4.079 (3.276-5.079) | 3.542 (3.369-3.725) |
| FPG HR at 5 years | 2.970 (2.149-4.105) | 2.790 (2.594-3.001) |

Both analyses used the same five-predictor M2 structure with FPG × log(t/3); only the coefficients were re-estimated in the expanded cohort. The FPG main-effect HR corresponds to the 3-year reference time. BMI, body mass index; DBP, diastolic blood pressure; FPG, fasting plasma glucose; β, regression coefficient; SE, standard error; HR, hazard ratio; CI, confidence interval.

**Supplementary Table 22. Discrimination and calibration of the complete-case and expanded five-predictor M2 parameter sets in a common internal validation cohort and external cohort.**

| **Cohort** | **Horizon, y** | **At risk / events** | **Parameter set** | **AUC** | **C-index** | **Brier** | **Observed risk** | **Predicted risk** | **O/E** |
| --- | --- | --- | --- | --- | --- | --- | --- | --- | --- |
| Expanded internal | 3 | 32,718 / 3,319 | Corrected complete-case | 0.771 | 0.762 | 0.060 | 0.069 | 0.077 | 0.898 |
| Expanded internal | 3 | 32,718 / 3,319 | Expanded five-predictor | 0.771 | 0.762 | 0.059 | 0.069 | 0.070 | 0.986 |
| Expanded internal | 4 | 14,481 / 5,641 | Corrected complete-case | 0.765 | 0.757 | 0.121 | 0.164 | 0.198 | 0.829 |
| Expanded internal | 4 | 14,481 / 5,641 | Expanded five-predictor | 0.766 | 0.757 | 0.119 | 0.164 | 0.169 | 0.967 |
| Expanded internal | 5 | 2,833 / 7,279 | Corrected complete-case | 0.737 | 0.752 | 0.208 | 0.343 | 0.427 | 0.803 |
| Expanded internal | 5 | 2,833 / 7,279 | Expanded five-predictor | 0.737 | 0.753 | 0.193 | 0.343 | 0.330 | 1.041 |
| External | 3 | 309 / 34 | Corrected complete-case | 0.748 | 0.721 | 0.065 | 0.075 | 0.082 | 0.910 |
| External | 3 | 309 / 34 | Expanded five-predictor | 0.745 | 0.717 | 0.065 | 0.075 | 0.074 | 1.004 |
| External | 4 | 122 / 59 | Corrected complete-case | 0.762 | 0.704 | 0.133 | 0.191 | 0.211 | 0.907 |
| External | 4 | 122 / 59 | Expanded five-predictor | 0.763 | 0.700 | 0.135 | 0.191 | 0.180 | 1.064 |
| External | 5 | 20 / 73 | Corrected complete-case | 0.666 | 0.700 | 0.226 | 0.425 | 0.452 | 0.940 |
| External | 5 | 20 / 73 | Expanded five-predictor | 0.669 | 0.699 | 0.234 | 0.425 | 0.349 | 1.219 |

Both parameter sets were evaluated in the same internal validation population and external cohort without recalibration. AUC and Brier scores were estimated using IPCW, and the C-index was calculated after administrative truncation at each horizon. Observed risk was estimated using the Kaplan–Meier method, and O/E was calculated as observed risk divided by mean predicted risk. AUC, area under the curve; IPCW, inverse-probability-of-censoring weighting; O/E, observed-to-expected ratio.

**Supplementary Table 23. Current-status interval-censoring sensitivity analysis: calibration and individual-level agreement with the final M2 model.**

| Cohort | Horizon, y | Turnbull observed risk, % (95% CI) | M2 predicted, % | M2 O/E | Interval-model predicted, % | Interval O/E | Spearman rho |
| --- | --- | --- | --- | --- | --- | --- | --- |
| Training set | 3 | 14.28 (12.85-15.43) | 8.38 | 1.70 | 13.96 | 1.02 | 0.958 |
| Training set | 4 | 14.28 (12.97-15.47) | 21.41 | 0.67 | 13.96 | 1.02 | 0.974 |
| Training set | 5 | 18.45 (16.05-23.19) | 45.22 | 0.41 | 20.33 | 0.91 | 0.956 |
| Internal validation set | 3 | 10.79 (9.48-12.33) | 8.30 | 1.30 | 13.89 | 0.78 | 0.958 |
| Internal validation set | 4 | 12.94 (10.55-16.42) | 21.21 | 0.61 | 13.89 | 0.93 | 0.974 |
| Internal validation set | 5 | 19.43 (15.77-25.66) | 44.92 | 0.43 | 20.24 | 0.96 | 0.956 |
| External cohort | 3 | 13.30 (9.57-16.81) | 8.10 | 1.64 | 13.55 | 0.98 | 0.955 |
| External cohort | 4 | 14.71 (10.68-21.05) | 20.85 | 0.71 | 13.55 | 1.09 | 0.971 |
| External cohort | 5 | 26.09 (13.84-45.03) | 44.57 | 0.59 | 19.84 | 1.31 | 0.951 |

Participants with an event were represented by the interval (0, follow-up time], whereas participants without an event were right-censored at follow-up. Turnbull estimates were used for observed risk, and O/E was calculated as observed risk divided by mean predicted risk. Spearman ρ represents agreement in individual-level risk ranking between the two models. This conservative representation was used because separate last-normal and first-abnormal examination times were unavailable. M2, the final extended Cox model; O/E, observed-to-expected ratio; CI, confidence interval.

**Supplementary Table 24. Time-specific associations of baseline FPG with incident prediabetes in the final extended Cox model (M2) and the current-status interval-censoring model.**

| Model | Time, y | FPG HR per mmol/L | 95% CI | Time function |
| --- | --- | --- | --- | --- |
| Final M2 extended Cox | 2 | 11.60 | 8.01-16.80 | log(t/3) |
| Final M2 extended Cox | 3 | 6.33 | 5.23-7.67 | log(t/3) |
| Final M2 extended Cox | 4 | 4.12 | 3.29-5.16 | log(t/3) |
| Final M2 extended Cox | 5 | 2.95 | 2.12-4.11 | log(t/3) |
| Current-status interval model | 2 | 2.94 | 2.24-3.87 | log((1+t)/4) |
| Current-status interval model | 3 | 2.01 | 1.16-3.48 | log((1+t)/4) |
| Current-status interval model | 4 | 1.49 | 0.67-3.30 | log((1+t)/4) |
| Current-status interval model | 5 | 1.17 | 0.43-3.18 | log((1+t)/4) |

M2 used log(t/3), whereas the current-status interval-censoring model used log[(1+t)/4] for the time-varying FPG effect. The interval-model analysis was exploratory because exact event intervals were unavailable and later-horizon estimates were imprecise. FPG, fasting plasma glucose; HR, hazard ratio; CI, confidence interval; M2, the final extended Cox model.

**Supplementary Table 25. Logistic regression additionally adjusted for follow-up duration in the training set.**

| Predictor | Adjusted OR | 95% CI | P value |
| --- | --- | --- | --- |
| Age, per year | 1.027 | 1.021-1.034 | <0.001 |
| BMI, per kg/m2 | 1.088 | 1.062-1.114 | <0.001 |
| DBP, per mmHg | 1.024 | 1.017-1.032 | <0.001 |
| FPG, per mmol/L | 4.310 | 3.515-5.309 | <0.001 |
| Family history, yes vs no | 1.611 | 1.214-2.115 | <0.001 |
| Follow-up duration, per year | 1.476 | 1.353-1.610 | <0.001 |

Follow-up duration was included as an additional covariate to assess the potential influence of unequal follow-up. This auxiliary logistic model was not used for survival or absolute-risk prediction. BMI, body mass index; DBP, diastolic blood pressure; FPG, fasting plasma glucose; OR, odds ratio; CI, confidence interval.

**Supplementary Table 26. Comparison of included and excluded participants.**

| Variable | Development set  (n = 10592) | Excluded set  (n = 174015) | P value |
| --- | --- | --- | --- |
| Male sex, n (%) | 6953 (65.6) | 91033 (52.3) | <0.001 |
| Smoking history, n (%) | 2480 (23.4) | 9346 (5.4) | <0.001 |
| Drinking history, n (%) | 2367 (22.3) | 6003 (3.4) | <0.001 |
| Family history of diabetes, n (%) | 630 (5.9) | 3042 (1.7) | <0.001 |
| FPG-defined incident prediabetes, n (%) | 1287 (12.2) | 19655 (11.3) | 0.007 |
| Age, years | 40.86±11.11 | 41.06±12.18 | 0.091 |
| BMI, kg/m² | 23.35±3.24 | 22.97±3.27 | <0.001 |
| SBP, mmHg | 117.97±14.92 | 117.84±15.88 | 0.395 |
| DBP, mmHg | 74.10±10.26 | 73.50±10.63 | <0.001 |
| FPG, mmol/L | 4.83±0.48 | 4.76±0.49 | <0.001 |
| Cholesterol, mmol/L | 4.71±0.88 | 4.66±0.89 | <0.001 |
| Triglyceride, mmol/L | 1.38±1.04 | 1.26±0.93 | <0.001 |
| HDL, mmol/L | 1.35±0.30 | 1.38±0.31 | <0.001 |
| LDL, mmol/L | 2.70±0.68 | 2.75±0.67 | <0.001 |
| ALT, U/L | 25.21±21.74 | 23.11±21.73 | <0.001 |
| AST, U/L | 24.64±10.44 | 23.57±12.69 | <0.001 |
| BUN, mmol/L | 4.69±1.16 | 4.60±1.17 | <0.001 |
| Creatinine, μmol/L | 73.38±15.24 | 69.42±15.73 | <0.001 |
| Follow-up duration, years | 3.05±0.90 | 3.15±0.94 | <0.001 |

Data are shown as means ± standard deviation for normally distributed variables and percentages for categorical variables. BMI, body mass index; SBP, systolic blood pressure; DBP, diastolic blood pressure; FPG, fasting plasma glucose; HDL, high-density lipoprotein; LDL, low-density lipoprotein; ALT, alanine aminotransferase; AST, aspartate aminotransferase; BUN, blood urea nitrogen.
